# Will *Casuarina glauca* Stress Resilience Be Maintained in the Face of Climate Change?

**DOI:** 10.3390/metabo11090593

**Published:** 2021-09-02

**Authors:** Tiago F. Jorge, José C. Ramalho, Saleh Alseekh, Isabel P. Pais, António E. Leitão, Ana P. Rodrigues, Paula Scotti-Campos, Ana I. Ribeiro-Barros, Alisdair R. Fernie, Carla António

**Affiliations:** 1 Plant Metabolomics Laboratory, Instituto de Tecnologia Química e Biológica António Xavier, Universidade Nova de Lisboa (ITQB NOVA), Avenida da República, 2780-157 Oeiras, Portugal; tiago.jorge89@gmail.com; 2 Plant Stress & Biodiversity Lab, Centro de Estudos Florestais (CEF), Instituto Superior de Agronomia (ISA), Universidade de Lisboa (ULisboa), Tapada da Ajuda, 1349-017 Lisboa and Quinta do Marquês, Portugal; cochichor@mail.telepac.pt (J.C.R.); antonioleitao@isa.ulisboa.pt (A.E.L.); anadr@isa.ulisboa.pt (A.P.R.); aribeiro@isa.ulisboa.pt (A.I.R.-B.); 3 GeoBioSciences, GeoTechnologies and GeoEngineering (GeoBioTec), Faculdade de Ciências e Tecnologia (FCT), Universidade NOVA de Lisboa (UNL), 2829-516 Monte de Caparica, Portugal; isabel.pais@iniav.pt (I.P.P.); paula.scotti@iniav.pt (P.S.-C.); 4 Central Metabolism Group, Max Planck Institute of Molecular Plant Physiology, 14476 Potsdam-Golm, Germany; alseekh@mpimp-golm.mpg.de (S.A.); fernie@mpimp-golm.mpg.de (A.R.F.); 5 Center of Plant Systems Biology and Biotechnology, 4000 Plovdiv, Bulgaria; 6 Unidade de Investigação em Biotecnologia e Recursos Genéticos, Instituto Nacional de Investigação Agrária e Veterinária, I.P. (INIAV), 2784-505 Oeiras, Portugal

**Keywords:** actinorhizal plants, *Casuarina glauca*, combined stress, heat stress, metabolomics, salt stress

## Abstract

Actinorhizal plants have been regarded as promising species in the current climate change context due to their high tolerance to a multitude of abiotic stresses. While combined salt-heat stress effects have been studied in crop species, their impact on the model actinorhizal plant, *Casuarina glauca*, has not yet been fully addressed. The effect of single salt (400 mM NaCl) and heat (control at 26/22 °C, supra optimal temperatures at 35/22 °C and 45/22 °C day/night) conditions on *C. glauca* branchlets was characterised at the physiological level, and stress-induced metabolite changes were characterised by mass spectrometry-based metabolomics. *C. glauca* could withstand single salt and heat conditions. However, the harshest stress condition (400 mM NaCl, 45 °C) revealed photosynthetic impairments due to mesophyll and membrane permeability limitations as well as major stress-specific differential responses in C and N metabolism. The increased activity of enzymatic ROS scavengers was, however, revealed to be sufficient to control the plant oxidative status. Although *C. glauca* could tolerate single salt and heat stresses, their negative interaction enhanced the effects of salt stress. Results demonstrated that *C. glauca* responses to combined salt-heat stress could be explained as a sum of the responses from each single applied stress.

## 1. Introduction

By the end of the 21st century, CO_2_ levels and global temperatures are expected to rise by 700 ppm and 1.5 °C, respectively [1]. Consequently, higher surface temperatures, frequent heat waves and intense precipitation events are likely to occur around the globe. These (stress)-factors negatively affect plant growth and development and trigger highly complex adaptive responses initiated by stress perception, signal transduction and the activation of stress-related genes and metabolites [2,3]. Most studies to date have focused on plant responses to a single stress and as such, do not mimic the natural environmental conditions wherein a combination of stresses is known to occur [4]. Recent studies have, however, demonstrated that combined stresses cannot be regarded as a sum of the responses induced from each single stress. In these studies, stress combinations elicited specific physiological, biochemical and metabolic responses due to a cross-talk of synergistic or antagonistic effects that are controlled by opposing signalling pathways [5,6,7,8,9]. Combined exposure to salt and heat stress has been shown to provide both positive and negative interactions in physiological traits [4,10,11,12]. In wheat (*Triticum aestivum* L. and *Triticum durum* Desf.), combined salt-heat stress promoted the uptake of toxic Na^+^ ions from salt stress and enhanced the heat stress-induced transpiration rate [13]. In tomato (*Solanum lycopersicum* L.), combined salt-heat stress ameliorated the effects of salt stress through the accumulation of glycine betaine and trehalose (well-known osmolytes), which helped improve plant performance by mitigating both photosynthetic damages and overaccumulation of reactive oxygen species (ROS) [14]. Single salt and heat stresses are known to trigger common molecular responses in plants [15]. An excess of Na^+^ ions in the soil can severely induce both osmotic and ionic stress within plant tissues leading to an impairment of the photosynthetic machinery and an accumulation of ROS that affect plant growth and metabolism [16,17]. Likewise, heat stress impairs the plant photosynthetic apparatus and metabolism due to a disruption of cell membrane fluidity that promotes overaccumulation of ROS and affects protein and enzyme stabilities [18,19]. Under single salt or heat stresses, plants were also reported to synthesize and accumulate osmolytes that play key roles in the regulation of osmotic potential and stabilization of biomolecules [20].

While the effects of a salt-heat stress combination have been mainly studied in crop species, their impact on ecologically important species has not yet been addressed. Actinorhizal plants are increasingly regarded as important species in the current climate change context [21,22] due to their ability to tolerate harsh environmental conditions [23,24]. This group of perennial dicotyledonous angiosperms establish root-nodule symbiosis with the N-fixation bacteria *Frankia* [24] and have a remarkable economic and ecological importance, often being used in the production of biomass and reclamation of degraded soils [25].

The model actinorhizal plant, *Casuarina glauca* Sieber (family *Casuarinaceae*), is native to Australia and widely distributed in areas where salt and heat stresses are likely to occur simultaneously (e.g., the Mediterranean, arid and semi-arid climates) [26,27]. The salt-stress tolerance of this species and the contribution of the symbiotic bacteria *Frankia Thr* strain have been thoroughly assessed at the physiological and biochemical levels [28,29,30,31,32,33,34,35,36]. These studies reported that, after exposing *C. glauca* Sieb. Ex Spreng nodulated (NOD^+^) and non-nodulated (KNO_3_^+^) plants up to 600 mM NaCl, an early salt-stress exposure (i.e., 200 mM NaCl) impairs the plant-bacteria symbiotic association [30]. Moreover, *C. glauca* plants withstand increasing salt concentrations by maintaining a controlled oxidative environment inside the cells and performing osmotic adjustments that contribute to a nearly stable photosynthetic machinery up to 400 mM NaCl [28,32]. These findings were supported by mass spectrometry (MS)-based proteomics studies that reported maintenance of the proteome stability at the highest salt-stress levels [36]. MS-based metabolomics studies revealed metabolite divergences in amino acid metabolism between NOD^+^ and KNO_3_^+^ plants and suggested that a flavonoid-based secondary antioxidant system complements ascorbate-glutathione cycle components that support the maintenance of a stable oxidative environment within the cell [33,35].

Given the highly promising tolerance of *C. glauca* to abiotic stresses, the effects of both single and combined exposure to salt and heat stress were investigated in *C. glauca* branchlets through physiological and MS-based metabolomics analyses in order to (i) evaluate the impact of combined salt-heat stress in the photosynthetic functioning (ii) characterise stress-induced metabolite changes, and (iii) identify key physiological and metabolite cross-responses associated with combined salt-heat stress in this species.

## 2. Results

### 2.1. Phenotypic Analysis and Plant Water Relations

**Figure 1:**
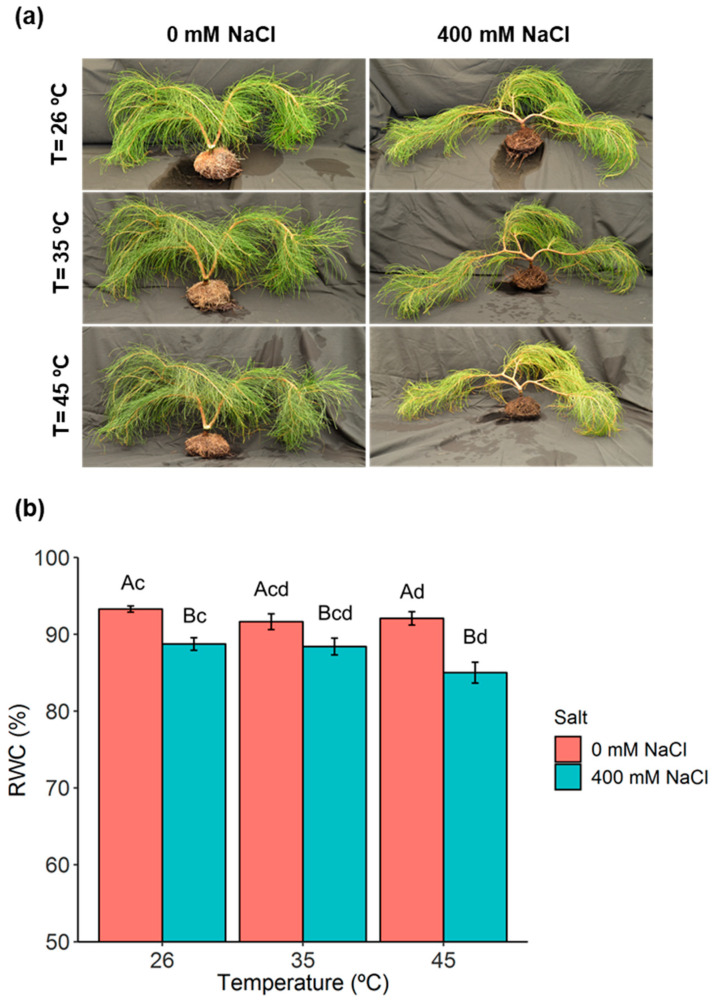
Evaluation of single salt, heat, and combined salt-heat stresses in *C. glauca* plants. (**a**) Visual evaluation of phenotypic changes in *C. glauca* plants under the single and combined exposure to salt (0 and 400 mM NaCl) and temperature (26, 35 and 45 °C) conditions. (**b**) Variation of the relative water content (RWC, %) in *C. glauca* branchlets under the single and combined exposure to salt (0 and 400 mM NaCl) and temperature (26, 35 and 45 °C) conditions. Bars represent the mean values ± SE from three to five independent measurements. Two-way ANOVA (*p* < 0.05) followed by Tukey’s HSD test was performed for means comparison, and different letters express significant differences between [NaCl] for the same temperature (A, B) or between temperature for the same [NaCl] (c, d), with A and c representing the highest values.

The effect of a single exposure to salt or heat stress conditions did not lead to major phenotypic changes in *C. glauca* plants. However, a pronounced visual phenotypic effect was observed at the harshest stress condition (400 mM NaCl, 45 °C) (Figure 1a). The impact of temperature on RWC was observed only at 45 °C, when compared to the control temperature (26 °C) and without salt exposure (Figure 1b). High salt concentration (400 mM NaCl) significantly decreased RWC values for all temperatures. This decrease was more pronounced at 400 mM NaCl, 45 °C; even when RWC values were reduced only to 85%.

### 2.2. Photosynthetic Gas Exchanges

**Figure 2:**
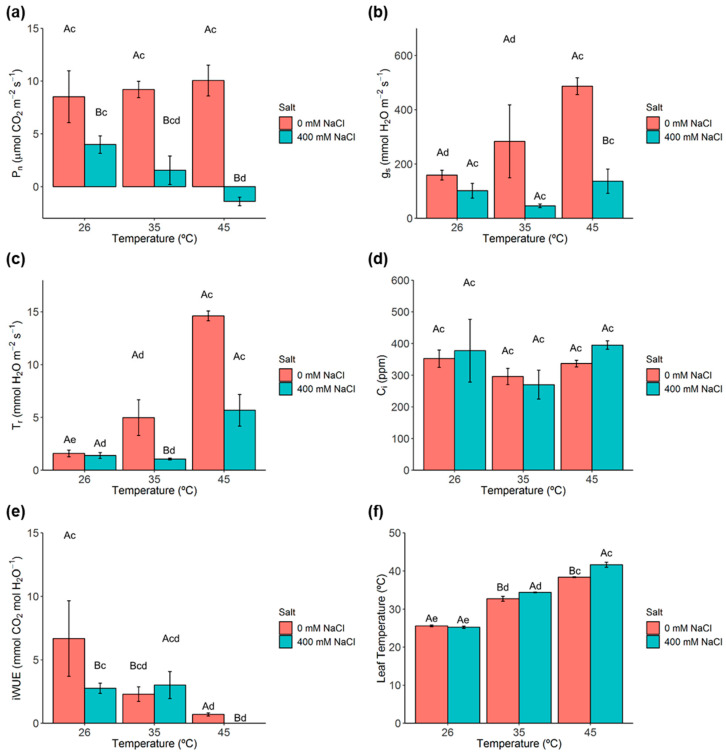
Analysis of gas exchange measurements in *C. glauca* branchlets under the single and combined exposure to salt (0 and 400 mM NaCl) and temperature (26, 35 and 45 °C) conditions. (**a**) Net photosynthetic rate (P_n_). (**b**) Stomatal conductance to water vapour rate (g_s_). (**c**) Transpiration rate (Tr). (**d**) Internal CO_2_ concentration (C_i_). (**e**) Instantaneous water use efficiency (iWUE). (**f**) Leaf temperature. Bars represent the mean values ± SE from three to five independent measurements. Two-way ANOVA (*p* < 0.05) followed by Tukey’s HSD test was performed for means comparison and different letters express significant differences between [NaCl] for the same temperature (A, B) or between temperature for the same [NaCl] (c, d, e) with A and c representing the highest values.

The net photosynthetic rate (P_n_) was not reduced with increasing temperature imposition, even at 45 °C. In contrast, P_n_ was significantly reduced under single salt conditions (26 °C), and was even more affected with the superimposition of heat, which led to negative values in plants exposed to 400 mM NaCl and 45 °C (Figure 2a).

Single heat stress (0 mM NaCl, 45 °C) induced a significant increase of stomatal conductance to water vapour rate (g_s_) (Figure 2b). However, under both high temperatures and salt conditions (35 and 45 °C, 400 mM NaCl), significant differences emerged between salt treatments mostly due to the large g_s_ increase in the 0 mM NaCl plants. Transpiration rate (Tr) followed a pattern close to that of g_s_, with significant increases under heat stress, while salt led to significant changes but only when combined with the highest temperatures (35 and 45 °C) (Figure 2c). Internal CO_2_ concentration (C_i_) did not statistically change under all treatments (Figure 2d), suggesting that the observed reduction of g_s_ in salt-exposed plants was not the main cause for the decline of P_n_ in these same plants. Regarding the instantaneous water use efficiency (iWUE), single exposure of salt, and heat led to significant reductions. However, a stronger iWUE reduction was observed under the simultaneous presence of both treatments (Figure 2e).

Significant increases in leaf temperature were promoted mainly by the rise in temperature (35 and 45 °C) as depicted from the similar patterns of 0 and 400 mM NaCl plants. The superimposition of salt led to significantly higher values of leaf temperature at 35 and 45 °C, likely related to the much lower Tr values in 400 mM NaCl plants as compared to 0 mM NaCl plants at those temperatures (Figure 2f).

### 2.3. Chlorophyll a Fluorescence

The impact of single and combined salt-heat stresses in the photosynthetic functioning was further evaluated through chlorophyll a fluorescence analysis (Table 1).

The maximal photochemical efficiency of PSII (F_v_/F_m_) was significantly affected only under the harshest stress condition (400 mM NaCl, 45 °C). The estimate of the quantum yield of non-cyclic electron transport (Y_(II)_) and the actual PSII photochemical efficiency (F_v_’/F_m_’) showed similar patterns of variation and decreased under both single stresses. This reduction was clearly stronger at 400 mM NaCl, 45 °C. The quantum yield of regulated energy dissipation of PSII (Y_(NPQ)_) significantly increased under salt conditions but only at 26 °C. Increasing temperatures also significantly increased Y_(NPQ)_ in the 400 mM NaCl plants. Consequently, Y_(NPQ)_ significantly increased at 400 mM NaCl, 45 °C. The quantum yield of non-regulated energy dissipation of PSII (Y_(NO)_) significantly increased under both single stresses, and consequently, strongly increased at 400 mM NaCl, 45 °C. The photochemical quenching based on the concept of separated (q_P_) antennae showed a similar pattern to that observed for Y_(II)_, F_v_’/F_m_’ with a statistically significant reduction at 400 mM NaCl, 45 °C. The total photoinhibition (PI_Tot_) significantly increased under both single stresses, but showed the highest modifications at 400 mM NaCl, 45 °C. Keeping in mind that Total PI is the sum of the chronic (PI_Chr_) and dynamic (PI_Dyn_) photoinhibitions it is highly relevant that the strong rise in Total PI reported at this condition was due almost entirely to a large increase in PI_Chr_.

### 2.4. Photosynthetic Pigments

Absolute levels for several photoprotective xanthophyll pigments, the de-epoxidation state (DEPS), and antioxidant carotenoids were next measured in *C. glauca* branchlets (Table 2 and Table S1). The levels of the xanthophyll cycle pigment violaxanthin (Viol) significantly decreased upon single salt stress with the concomitant synthesis of antheraxanthin (Ant) and zeaxanthin (Zea), which increased (also under heat conditions). As for the photoprotective (dissipative) Ant and Zea, the highest increases were observed under the harshest stress condition (400 mM NaCl, 45 °C). The de-epoxidation state (DEPS) was significantly increased by both single stresses, but particularly when they were combined. Lutein levels significantly decreased under salt conditions but significantly increased due to heat, without apparent interaction of these stress conditions. The levels of β-Carotene significantly decreased under salt stress conditions at 35 °C, whereas at higher temperatures (35 and 45 °C) and 0 mM NaCl, its levels significantly increased. The harshest stress condition led to a significant increase of β-Carotene in comparison with control conditions (0 mM NaCl, 26 °C).

### 2.5. Ribulose-1,5-Biphosphate Carboxylase/Oxygenase (RuBisCO) Activity and Activation State

**Figure 3:**
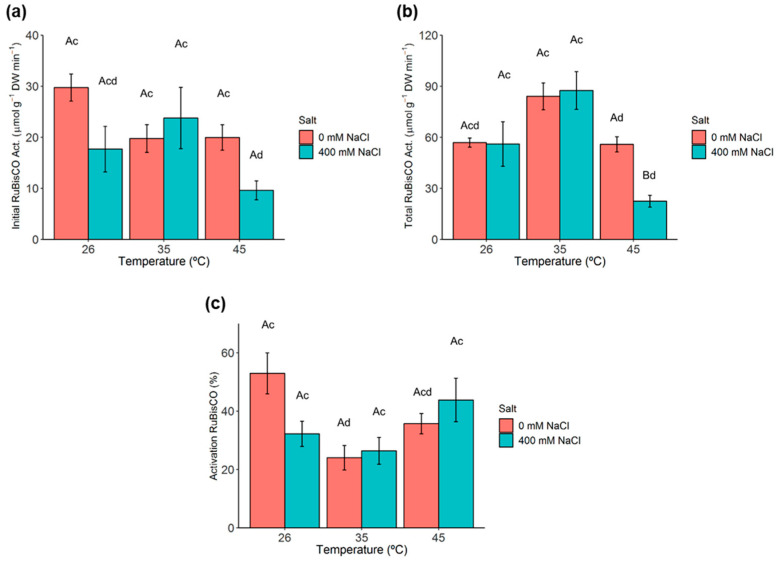
Variation of the ribulose-1,5-biphosphate carboxylase/oxygenase (RuBisCO) activity and activation state in *C. glauca* branchlets under the single and combined exposure to salt (0 and 400 mM NaCl) and temperature (26, 35 and 45 °C) conditions. (**a**) Initial activity. (**b**) Total activity. (**c**) Activation state. Bars represent the mean values ± SE from three to five independent measurements. Two-way ANOVA (*p* < 0.05) followed by Tukey’s HSD test was performed for means comparison and different letters express significant differences between [NaCl] for the same temperature (A, B) or between temperature for the same [NaCl] (c, d) with A and c representing the highest values.

The initial activity of RuBisCO was not significantly impacted by both single stresses (Figure 3a). Notably, despite the total activity of RuBisCO did not significantly increase at 35 °C (Figure 3b), a significant reduction in the activation status in the 0 mM NaCl plants at this temperature was observed (Figure 3c). Indeed, only at the harshest stress condition (400 mM NaCl, 45 °C) were clear reductions in both activities observed. However, even under these conditions, the activation state remained unaltered.

### 2.6. Membrane Permeability and Lipid Peroxidation

**Figure 4:**
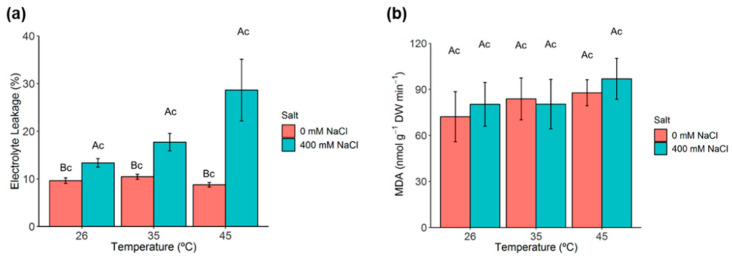
Membrane permeability and lipid peroxidation in *C. glauca* branchlets under the single and combined exposure to salt (0 and 400 mM NaCl) and temperature (26, 35 and 45 °C) conditions. (**a**) Electrolyte leakage. (**b**) Malonyldialdehyde (MDA) content. Bars represent the mean values ± SE from three to five independent measurements. Two-way ANOVA (*p* < 0.05) followed by Tukey’s HSD test was performed for means comparison, and different letters express significant differences between [NaCl] for the same temperature (A, B) or between temperature for the same [NaCl] (c) with A and c representing the highest values.

Membrane permeability and lipid peroxidation were assessed in *C. glauca* branchlets through electrolyte leakage and malonyldialdehyde (MDA) analyses, respectively. The percentage of electrolyte leakage significantly increased under salt conditions (Figure 4a). The MDA content did not change significantly under any salt or temperature treatment (Figure 4b).

### 2.7. Lipid Analyses

Total fatty acid (TFA) content, individual fatty acid (FA) composition, and the unsaturation (DBI) of total lipids were measured in *C. glauca* branchlets (Table 3). Only those demonstrated to significantly change in at least one condition are described.

Salt led to a significant decrease in the TFA content, while temperature did not change this parameter. Similarly, high salt concentration (400 mM NaCl) and high temperature (45 °C) significantly decreased the amount of linolenic acid (C18:3), with the latter one only at 400 mM NaCl. By contrast, both single stresses significantly increased linoleic acid (C18:2); however, this increase was more evident under salt conditions. Likewise, palmitic acid (C16:0) and oleic acid (C18:1) significantly increased under salt conditions (only at 45 °C). Additionally, increasing temperatures led to a significant increase of C16:0 and C18:1; however, for C16:0, this increase was only significant at 400 mM NaCl. Oppositely, both single stresses significantly decreased DBI at the harshest stress condition (400 mM NaCl, 45 °C).

### 2.8. Antioxidant Enzymes

**Figure 5:**
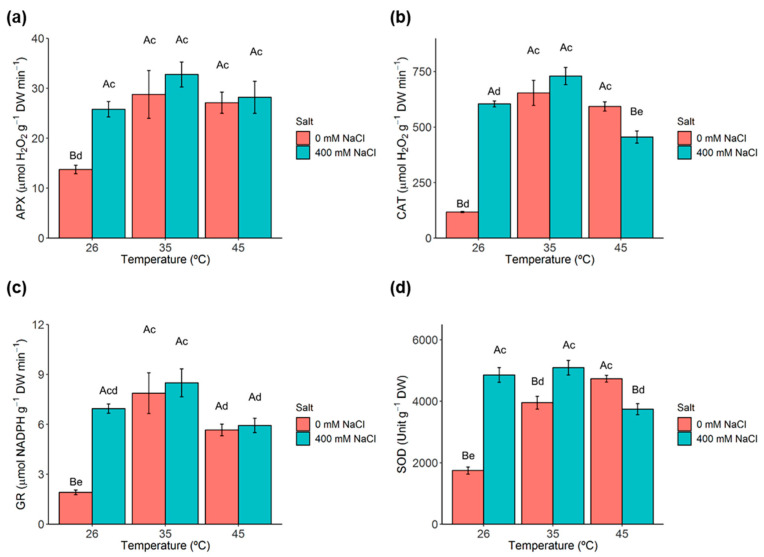
Evaluation of antioxidant enzyme activities in *C. glauca* branchlets under the single and combined exposure to salt (0 and 400 mM NaCl) and temperature (26, 35 and 45 °C) conditions. (**a**) ascorbate peroxidase (APX). (**b**) cellular catalase (CAT). (**c**) glutathione reductase (GR). (**d**) superoxide dismutase (SOD). Bars represent the mean values ± SE from three to five independent measurements. Two-way ANOVA (*p* < 0.05) followed by Tukey’s HSD test was for performed for means comparison and different letters express significant differences between [NaCl] for the same temperature (A, B) or between temperature for the same [NaCl] (c, d, e) with A and c representing the highest values.

Both single and combined exposure to salt and heat stress significantly increased ascorbate peroxidase (APX) activity in *C. glauca* branchlets (Figure 5a). A similar pattern of variation was observed for catalase (CAT), in which single salt and heat conditions significantly increased CAT activity up to, and including, 35 °C. The harshest stress condition (400 mM NaCl, 45 °C) significantly decreased CAT activity with respect to the levels at 35 °C, although to levels higher than the control (0 mM NaCl, 26 °C) (Figure 5b). Glutathione reductase (GR) activity also significantly increased under single salt and heat conditions, with the most pronounced effect of salt observed at the lowest temperature (26 °C). The highest temperature (45 °C) resulted in significantly decreased GR activity at both control (0 mM NaCl) and 400 mM NaCl (Figure 5c). Similarly, superoxide dismutase (SOD) activity significantly increased under both salt (at 26 and 35 °C) and heat (only at 0 mM NaCl) conditions. By contrast, at 45 °C, salt conditions decreased SOD activity; however, such decrease did not reduce SOD activity at the harshest stress conditions (Figure 5d).

### 2.9. GC-TOF-MS Primary Metabolite Profiling Analysis

**Figure 6:**
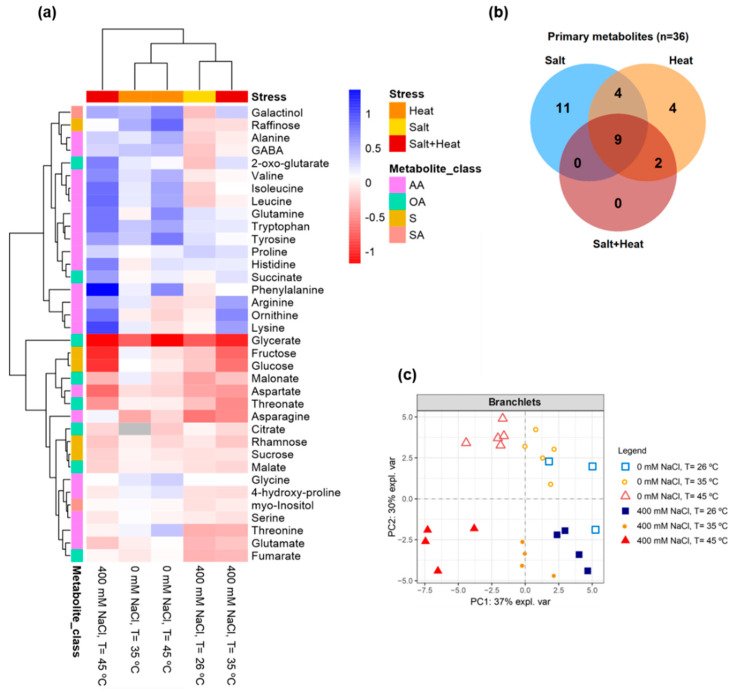
GC-TOF-MS primary metabolite profiling analysis. (**a**) Heatmap showing metabolite responses in *C. glauca* branchlets under the single and combined exposure to salt (0 and 400 mM NaCl) and temperature (26, 35 and 45 °C) conditions. Relative values are normalised to the internal standard (ribitol) and dry weight (DW) of the samples. False-colour imaging was performed on Log_10_-transformed GC-TOF-MS data. Grey-colour squares represent not detected (n.d.) values. AA-amino acids, OA-organic acids, S-sugars, SA-sugar alcohols. (**b**) Venn diagram showing the number of metabolites which levels were shown to significantly change in *C. glauca* branchlets under the single and combined exposure to salt (0 and 400 mM NaCl) and temperature (26, 35 and 45 °C) conditions. Two-way ANOVA (*p* < 0.05) followed by Tukey’s HSD test was performed for means comparison. (**c**) Principal component analysis (PCA) score plots of the primary metabolite profiles in the branchlets of *C. glauca* plants under the single and combined exposure to salt (0 and 400 mM NaCl) and temperature (26, 35 and 45 °C) conditions.

Primary metabolite profiling analysis allowed the detection of 36 metabolites in *C. glauca* branchlets (Figure 6a, Table S2). Under salt stress, two-way ANOVA analysis revealed that the relative levels of 11 primary metabolites significantly changed (fructose, glucose, rhamnose, sucrose, glutamate, proline, 4-hydroxy-proline, aspartate, malate, threonate and malonate) as shown in the Venn diagram (Figure 6b, Table S2). Of these, only proline significantly increased (2-fold), whereas fructose, glucose, rhamnose, sucrose, glutamate, 4-hydroxy-proline, aspartate, malate, threonate and malonate significantly decreased. By contrast, heat stress significantly changed the levels of the amino acids tryptophan, tyrosine, valine and glutamine (Figure 6a,b, Table S2). All these metabolites significantly increased at 35 °C (up to 2-fold) and 45 °C (up to 6-fold). On the other hand, galactinol, alanine, γ-aminobutyric acid (GABA) and fumarate were shown to significantly change by both single stresses, but not by combined salt-heat stress (Figure 6a,b, Table S2). These metabolites significantly decreased at 400 mM NaCl, and significantly increased under increasing temperatures of 35 and 45 °C (up to 5-fold). Lastly, single heat and combined salt-heat stresses significantly changed the levels of isoleucine and leucine, which increased up to 3-fold (heat stress) and up to 7-fold (salt-heat stress) (Figure 6a,b, Table S2).

Under both single and combined salt-heat stresses, nine primary metabolites were shown to significantly change; namely, raffinose, glycine, phenylalanine, histidine, ornithine, arginine, lysine, threonine and glycerate (Figure 6a,b, Table S2). Of these, the most representative significant increases were found for raffinose (up to 7-fold, heat stress), glycine (up to 2-fold, heat stress), phenylalanine (up to 5-fold, heat stress; up to 22-fold, salt-heat stress), histidine (up to 5-fold, salt-heat stress), ornithine (up to 6-fold, salt-heat stress), arginine (up to 4-fold, salt-heat stress), lysine (up to 10-fold, salt-heat stress) and threonine (up to 2-fold, heat stress), while significant decreases were observed for raffinose (salt stress), ornithine (heat stress), threonine (salt stress) and glycerate (for all stresses).

PCA revealed that salt treatments (0 and 400 mM NaCl) were separated along with the principal component 2 (PC2) (Figure 6c).

Regarding temperature conditions, it was observed that samples at 45 °C were clearly separated from control samples (26 °C) by the principal component 1 (PC1).

**Figure 7:**
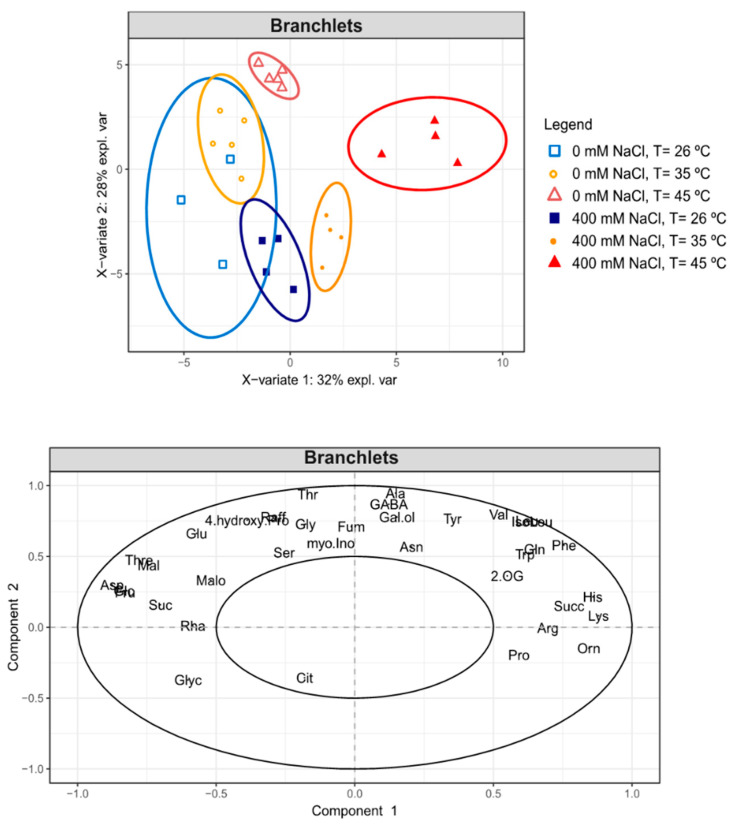
Partial least square discriminant analysis (PLS-DA) score and loading plot in the branchlets of *C. glauca* plants under the single and combined exposure to salt (0 and 400 mM NaCl) and temperature (26, 35 and 45 °C) conditions.

Similarly, partial least square discriminant analysis (PLS-DA) score plot revealed a clear separation of the different heat treatments, more evident for the salt-treated plants between samples at 26 and 45 °C (Figure 7). A different behaviour was observed between salt samples (0 versus 400 mM NaCl) at 26 °C, which did not separate. By contrast, the harshest stress condition (400 mM NaCl, 45 °C) was clearly separated from all the other conditions. The PLS-DA loading plot confirmed that the metabolites that statistically increased under both single and combined salt-heat stresses were also responsible for the discrimination of the different sample groups; phenylalanine, histidine, ornithine, arginine and lysine being clearly responsible for the separation of the harshest stress condition (400 mM NaCl, 45 °C) from the control condition (0 mM NaCl, 26 °C) (Figure 7, Table S2). 

### 2.10. LC-HRMS/MS Target Secondary Metabolite Analysis

Target secondary metabolite analysis allowed the detection of 23 metabolite features in *C. glauca* branchlets, 16 of which were identified based on their product ion spectra and mass spectral databases (Figure S2, Tables S3 and S4). In particular, flavanols, mainly kaempferol and quercetin glycosides, were the major metabolites identified. Among them, kaempferol-*O*-glycosides [(kaempferol 7-*O*-alpha-rhamnoside-D-glucoside, *m/z* = 593 [M-H]^−^; kaempferol 3-*O*-(6″-galloyl)-beta-D-glucopyranoside, *m/z* = 599 [M-H]^−^; kaempferol-3-*O*-rhamnoside, *m/z* = 431 [M-H]^−^; two kaempferol-3,7-*O*-bis-alpha-L-rhamnoside, *m/z* = 577 [M-H]^−^)] and quercetin-*O*-glycosides [(quercetin-3-*O*-arabinoside, *m/z* = 431 [M-H]^−^; quercetin-3-*O*-(6″-*O*-galloyl)-beta-galactoside, *m/z* = 615 [M-H]^−^; quercetin-3-*O*-glucuronide, *m/z* = 477 [M-H]^−^; quercetin-7-*O*-rhamnoside, *m/z* = 447 [M-H]^−^)] were identified (Figure S2, Table S3). Anthocyanins [malvidin-3-*O*-galactoside; cyanidin-3-*O*-(6-*O*-p-coumaryl)-pentoside], gallic-acid derivatives [methyl 6-*O*-galloyl-beta-D-glucopyranoside (*m/z* = 345 [M-H]^−^); methyl 4,6-di-*O*-galloyl-beta-D-glucopyranoside (*m/z* = 497 [M-H]^−^)] and complex ellagitannin polymeric structures [pedunculagin and two of its isomers (*m/z* = 783 [M-H]^−^); casuarictin (*m/z* = 935 [M-H]^−^) and two of its isomers] were also identified (Figure S2, Table S3).

The majority of the identified secondary metabolites showed to decrease under single heat conditions while their levels were differentially changed under salt conditions (Figure S2, Table S4). The harshest stress condition (400 mM NaCl, 45 °C) increased the levels of almost all detected secondary metabolites. Subsequent two-way ANOVA analysis revealed that only kaempferol-3-*O*-rhamnoside, unknown_1 and kaempferol-3,7-*O*-bis-alpha-L-rhamnoside significantly increased only by single salt stress (Table S4).

PCA did not reveal major trends among treatments (Figure S3). Subsequent PLS-DA revealed a pattern close to that of PCA (Figure S4). Indeed, the PLS-DA score plot revealed that almost all conditions clustered together. A minor discrimination between the control condition (0 mM NaCl, 26 °C) and the harshest stress condition (400 mM NaCl, 45 °C) was observed. The corresponding contribution plot revealed that most of the detected metabolites contributed to this discrimination. Indeed, the metabolites that showed to significantly change by two-way ANOVA (kaempferol-3-*O*-rhamnoside, unknown_1 and kaempferol-3,7-*O*-bis-alpha-L-rhamnoside) were the metabolites that mostly contributed to this separation.

## 3. Discussion

Single salt or heat stress promotes the development of a water deficit status within the cell [18,37,38]. In *C. glauca* branchlets, the observed significant decrease of RWC upon salt stress is consistent with previous reports in *C. glauca* NOD^+^ and KNO_3_^+^ plants submitted to salt concentrations up to 600 mM NaCl [28]. Gas exchanges (P_n_, g_s_, Tr) were not negatively affected by heat stress in *C. glauca*, in line with the known environmental resilience of these plants [31]. However, these parameters were impacted under single salt stress. Interestingly, the harshest stress condition (400 mM NaCl, 45 °C) resulted in a strong impairment in P_n_, which became negative. This reduction in P_n_ is likely to be independent of a stomata limitation since C_i_ did not change significantly, suggesting that a CO_2_ supply to the carboxylation sites was not limited [39]. With this observation being in keeping with previous results reported for *C. glauca* KNO_3_^+^ plants submitted to 400 mM NaCl [28]. The significant increase of both g_s_ and Tr furthermore suggests that plants neither limit stomata opening nor their rates of transpiration.

Likewise, in tomato plants, combined salt-heat stress led to a higher increase of Tr in comparison to that observed for salt stress alone [14].

Indeed, single stress alone did not affect the photosynthetic functioning in *C. glauca* branchlets. However, under the harshest stress condition, mesophyll limitation of photosynthesis resulted from impairments in RuBisCO and PSII, presumably promoted by the increase in temperature. At the PSII level, the harshest stress condition evoked decreases in widely used photochemical indicators of overall photosynthetic function [40]. The quantum yield of non-regulated energy dissipation of PSII(Y_(NO)_) denoted the highest rise, in line with the higher photoinhibitory status suggested by the large increase in chronic (PI_Chr_) and total (_t_ Total PI) photoinhibitions. Y_(NO)_ reflects the photoinactivation and non-regulated energy dissipation in PSII (heat and fluorescence) [41,42,43]. Y_(NO)_ is mostly stable, even under stressful conditions [42], with a high value pointing to constraints in radiation-energy use due to a lack of both quantum yield of non-cyclic electron transport (Y_(II)_) and of regulated energy dissipation of PSII (Y_(NPQ)_) [43,44].

Despite the above-mentioned negative impacts, some mechanisms of controlled energy dissipation (q_N_, Y_(NPQ)_, Zea, lutein, β-Carotene) that are well known to be crucial in the photoprotection of PSII were activated in *C. glauca* under stress conditions (Maxwell and Johnson, 2000 [40,45,46,47,48]. However, results show that under strict stress conditions, the triggered protective mechanisms did not prevent a significant impact at the photochemical efficiency of PSII and the biochemical (RuBisCO) performance levels.

Single salt and heat stresses are known to induce structural changes in the cellular membrane that alter its permeability, composition and fluidity [49,50]. Damages to cellular membranes are often associated with an overaccumulation of ROS that eventually induces the lipoperoxidation of membrane constituents, namely polyunsaturated fatty acids [51,52,53].

Single salt conditions (400 mM NaCl) significantly increased electrolyte leakage in *C. glauca* branchlets. Indeed, it was previously demonstrated that increasing salt concentrations (400 mM NaCl onwards) damaged membrane permeability in *C. glauca* NOD^+^ and KNO_3_^+^ plants [28]. On the other hand, any of the heat stress conditions changed membrane permeability, which indicates the ability of *C. glauca* to tolerate high temperatures. Nevertheless, at 400 mM NaCl, 45 °C, membrane permeability was severely impaired, whereas MDA levels remained quite stable under all stress conditions. The latter demonstrates that the oxidative degradation of membrane lipids was not triggered by any of these stress conditions. These results agree with the reported maintenance of MDA levels under increasing salt concentrations (up to 600 mM NaCl) in *C. glauca* plants [32].

The impact of single salt stress on TFA and individual FA composition (with the exception of C16:1 and of less representative FAs) showed similar results to those previously reported for *C. glauca* KNO_3_^+^ plants up to 400 mM NaCl [32]. In the case of C18:3, the most abundant FA in chloroplast membranes (ca. 70%) [54], its levels significantly decreased under all stress conditions (more pronounced at 400 m M NaCl, 45 °C). Indeed, decreased levels of C18:3 (with simultaneous increase of C18:1 levels) were associated to high-temperature tolerance in plants, whereas the same pattern is commonly associated to impairments of cellular membranes under salt stress conditions [51,55]. By contrast, C16:0 was found to significantly increase in *C. glauca* branchlets at 400 mM NaCl, 45 °C, while DBI levels were strongly reduced. Altogether, these data indicate the ability of *C. glauca* to adjust FA unsaturation levels under stress conditions while maintaining constant percentages of MDA. FA modifications, together with the impairment of the membrane permeability, likely indicate damages at the membrane levels, and agrees with the impacts on the photosynthetic functioning in PSII, at the harshest stress condition.

PCA revealed that both single and combined salt-heat stresses induced differential metabolite responses. Subsequent two-way ANOVA and PLS-DA analyses allowed identifying as stress-responsive metabolites (i.e., metabolites that significantly accumulated): (i) proline (for single salt stress), (ii) raffinose, galactinol, glycine, phenylalanine, tryptophan, tyrosine, alanine, valine, GABA, glutamine, threonine, isoleucine, leucine and fumarate (for single heat stress), (iii) phenylalanine, histidine, ornithine, arginine and lysine (single salt, heat and combined salt-heat stresses). Particular attention is given below to some of these metabolites.

Single heat stress significantly increased the levels of galactinol and raffinose; galactinol being one main precursor of the raffinose family oligosaccharides (RFOs) that include raffinose. These molecules are known to accumulate in plants under stress conditions where they act as osmoprotectants [56,57].

Amino acids constituted the major metabolite class identified in *C. glauca* branchlets. Amongst them, glycine levels were shown to significantly increase upon both single and combined salt-heat stresses (more pronounced under heat stress). Glycine is a photorespiratory amino acid, and this result might provide further support for an increased rate of the photorespiration pathway [58,59,60] under both single and combined salt-heat stresses.

Amino acids derived from the shikimate pathway; namely, phenylalanine, tryptophan and tyrosine accumulated under heat stress (phenylalanine also increased under combined salt-heat stress). Overall, the accumulation of these metabolites can be a useful source of C skeletons for the phenylpropanoid pathway and biosynthesis of secondary metabolites [61,62], but their accumulation is also tightly linked to the operation of the Calvin-Benson cycle, with shikimate derived amino acids being the most rapidly labelled following feeding of *Arabidopsis* or maize with ^13^CO_2_ [63,64].

Branched-chain amino acids; namely, valine, isoleucine and leucine have also been reported to accumulate under abiotic stress conditions [65,66]. Similarly, here we reported increased levels of valine (45 °C) and higher levels of leucine and isoleucine under single heat and combined salt-heat stresses in *C. glauca* branchlets.

GABA, proline and ornithine are some of the amino acids derived from 2-oxo-glutarate (2-OG); an intermediate of the mitochondrial tricarboxylic acid (TCA) cycle. An increase in the levels of metabolites involved in the GABA shunt (glutamate, GABA, alanine) was suggested to act as an intermediate supplier to feed the TCA cycle during heat stress and help maintain metabolic homeostasis [67]. Indeed, the GABA shunt is known to play a role in the regulation of C and N metabolism [68,69]. Proline significantly increased under salt stress, which agrees with its well-known osmoprotectant role in plants, including salt stress [70,71,72,73]). Ornithine is one of the most abundant amino acids in *Casuarina* plants and has been suggested to play a role as an intermediate metabolite in N transport [74]. This metabolite was previously reported as a salt-stress responsive metabolite in *C. glauca* plants [33]. Accordingly, ornithine levels significantly increased under single salt and combined salt-heat stresses.

Threonine and lysine comprise the aspartate-family amino acids derived from the TCA-cycle intermediate oxaloacetate (OAA). Lysine significantly increased under single salt, single heat and combined salt-heat stresses; increased levels of lysine, and subsequent decrease in the levels of TCA-cycle metabolites, have been reported in plants subjected to stresses that lead to energy deprivation [75]. Indeed, fumarate significantly decreased upon single salt stress, as previously observed in *C. glauca* tissues [33].

Single salt and heat stresses are also known to induce oxidative stress inside plant cells [71,76]. An overaccumulation of ROS causes oxidative damage to cell membranes leading to lipid peroxidation, photosynthetic damages and DNA degradation [77,78]. Increased activity of enzymatic ROS scavengers; namely, APX, CAT, GR and SOD reflects an oxidative environment inside the cells [53]. In *C. glauca* branchlets, APX, CAT, GR and SOD significantly increased under single salt stress. The activity of these enzymes was previously demonstrated to increase in *C. glauca* plants upon early salt stress conditions (i.e., 200 mM NaCl) [32]. Single heat and combined salt-heat stresses also significantly increased the activity of these enzymes; however, this increase was not so pronounced at the highest temperature (45 °C). These results likely contribute to the absence of lipoperoxidation under the studied stress conditions.

Important non-enzymatic ROS scavengers, including flavonoids (flavones and flavonols) have been regarded as being part of a secondary antioxidant system that complements the action of enzymatic ROS scavengers [53,79].

LC-HRMS/MS target secondary metabolite analysis in *C. glauca* branchlets under both single and combined salt-heat stresses allowed detecting kaempferol-*O*-glycosides and quercetin-*O*-glycosides. Two-way ANOVA analysis, together with the observed overlapping of the studied stress conditions in the PLS-DA, likely suggests that *C. glauca* branchlets did not induce the activation of a non-enzymatic ROS scavenger flavonoid-based secondary antioxidant system.

## 4. Materials and Methods

### 4.1. Plant Growth Conditions and Stress Treatments

Approximately 100 needle-like branchlets (8–10 cm long) of a 2-year old *Casuarina glauca* Sieb. Ex Spreng. plant was used for vegetative propagation in Broughton and Dillworth’s (BD) modified medium [80] after immersion in 50 ppm of indole butyric acid (IBA) for 24 h [81]. 1-year old *C. glauca* plants were transferred into a walk-in growth chamber (10000 EHHF, ARALAB, Portugal) under environmental controlled conditions of photoperiod (12 h), temperature (26/22 °C, day/night), relative humidity (70%), and irradiance (ca. 500 µmol m^−2^ s^−1^). Plants were divided in 2 groups and submitted to 0 mM (control) and 400 mM NaCl conditions. Salt enhancement in the hydroponic nutrient solution [(BD) modified medium] was gradually imposed (50 mM NaCl week^−1^) to avoid osmotic shock and to allow the plants to express their potential for acclimation. Plants were kept for 1 more week at 400 mM NaCl before the temperature treatment began. Two plant groups were gradually submitted to a diurnal temperature rise (1 °C day^−1^) to avoid heat shock, from 26 °C (control) to 45 °C (Figure S1). Measurements were performed at 26, 35 and 45 °C. Plants were kept for 5 days at 35 and 45 °C.

### 4.2. Harvest and Storage of Casuarina Glauca Branchlets

*C. glauca* branchlets were harvested at each temperature during the same daylight period after ca. 2 h (10–12 h) of irradiance (*ca.* 500 µmol m^−2^ s^−1^), immediately frozen in liquid nitrogen, and stored at −80 °C until further analyses. Three to five independent plants in the vegetative developmental stage were used per independent treatment.

### 4.3. Plant Water Relations

Relative water content (RWC) measurements were performed as previously described in [28]. RWC was determined at midday using 9 *C. glauca* branchlet pieces (1.5–2.0 cm long) per biological replicate.

### 4.4. Gas Exchange Measurements and Chlorophyll a Fluorescence

Gas exchanges were determined following [82]. The rates of branchlet net photosynthesis (P_n_), stomatal conductance to water vapour rate (g_s_), transpiration rate (Tr), internal CO_2_ concentration (C_i_) and leaf temperature were assessed at each temperature condition under photosynthetic steady-state conditions after ca. 2 h (10–12 h) of irradiance (*ca.* 500 µmol m^−2^ s^−1^) using a CO_2_/H_2_O portable open-system infrared gas analyser (CIRAS 1, PP Systems,Amesbury, MA, USA).

Chlorophyll (Chl) *a* fluorescence parameters were determined on the same *C. glauca* branchlets used for the gas exchange measurements using a PAM-2000 system (H. Walz, Effeltrich, Germany) as previously described [28,44], and applying the formulae discussed elsewhere [41,83,84]. Measurements of the minimal fluorescence from antennae (F_o_), maximal fluorescence of the primary PSII (F_m_), and maximal photochemical efficiency of PSII (F_v_/F_m_) were performed overnight dark-adapted *C. glauca* branchlets.

Another set of parameters was evaluated under photosynthetic steady-state conditions, using ca. 510 μmol m^−2^ s^−1^ of actinic light and superimposed saturating flashes: q_P_, q_L_, NPQ, Y_(II)_, Y_(NPQ)_, Y_(NO)_, F_v_′/F_m_′ and PSII photoinhibition indexes (For more details, see Methods S1).

### 4.5. Photosynthetic Pigment Measurements

Carotenoids were assessed from 2-3 *C. glauca* branchlet pieces FW (*ca.* 50 mg). Sample processing and subsequent reverse-phase HPLC analysis were carried out as described in [85] using an end-capped (C_18_) 5 μm Spherisorb ODS-2 column (250 × 4.6 mm). Detection was performed at 440 nm in an HPLC system (Beckman, System Gold, Tulsa, OK, USA) coupled to a diode-array detector (Model 168; Beckman, Tulsa, OK, USA). Identification and quantification were performed using individual authentic standards (Merck Life Science S.L., PT).

### 4.6. Ribulose-1,5-Bisphosphate Carboxylase/Oxygenase Activity

Ribulose-1,5-bisphosphate carboxylase/oxygenase (RuBisCO: EC 4.1.1.39) enzymatic activities were determined in ca. 200 mg FW of frozen *C. glauca* branchlet tissues. Each sample was homogenised in a pre-cooled pestle and mortar using 100 mg of insoluble polyvinylpyrrolidone (PVPP) and 1 mL of extraction buffer 100 mM Tris-HCl (pH 8) containing 10 mM MgCl_2_, 15 mM NaHCO_3_, 10 mM β-mercaptoethanol, 2 mM ditiotreitol (DTT), 1% (*v/v*) Triton X-100, 10% (*v/v*) glycerol and 2% (*v/v*) of a cOmplete™ EDTA-free Protease Inhibitor. Extracts were centrifuged (16,000× *g*, 20 min, 4 °C) and the supernatant was used for enzyme assays. The initial and total activities of RuBisCO were determined following the 3-PGA-dependent NADH oxidation at 340 nm and 25 °C, in a final volume of 1 mL [86].

### 4.7. Electrolyte Leakage

Membrane permeability was determined using 9 branchlet pieces (*ca.* 1.5 cm), rinsed 3 times, and subsequently floated in flasks containing 15 mL of demineralized water. Water conductivity was measured using a conductimeter (Crison GLP31, Crison Instruments, S.A., Spain) following a 24 h period of floating at ca. 20 °C as described previously [87]. Total conductivity was obtained after keeping the flasks in an oven at 90 °C for 2 h, followed by cooling down to 20 °C. Electrolyte leakage was expressed as the percentage of total conductivity.

### 4.8. Lipid Analyses

Lipid components of cellular membranes were determined as previously described [88]. Briefly, ca. 1 g FW frozen *C. glauca* branchlet tissues were boiled for 2 min in distilled water to stop lipolytic activities. Total lipids were extracted in a mixture of chloroform/methanol/water (1/1/1; *v/v/v*). For fatty acid (FA) analysis, aliquots of total lipid extracts were saponified. Heptadecanoic acid (C17:0) was added as internal standard, followed by methyl esterification with trifluoroborane (BF3)-methanol. Two methylation replicates per sample were performed for each extract. Fatty acid methyl esters were analysed with GC-FID (CP-3380, Varian, CA, USA). For more details, see Methods S2.

### 4.9. Antioxidant Enzyme Activities

Antioxidant enzyme activities were determined from 1 g FW frozen *C. glauca* branchlet tissues. For superoxide dismutase (SOD; EC 1.15.1.1) and glutathione reductase (GR; EC1.6.4.2) activities, branchlet tissue was homogenised in 1.0 mL of 100 mM sodium phosphate buffer (pH 7.8) containing 1% of polyvinylpyrrolidone (PVPP) and centrifuged (10,000× *g*, 15 min, 4 °C). The supernatant was used for the estimation of SOD and GR. SOD activity was estimated following the enzyme activity that inhibited the photoreduction of NBT to blue formazan by 50% [89]. GR activity was measured following the absorbance decrease at 340 nm corresponding to the NADPH oxidation rate [90].

For catalase (CAT; EC 1.11.1.6), branchlet tissue was homogenised in 1.0 mL of 100 mM sodium phosphate buffer (pH 7.0) containing 1% PVPP followed by centrifugation (10,000× *g*, 20 min, 4 °C). The supernatant was used for the estimation of CAT. CAT activity was estimated based on the decrease in absorbance of H_2_O_2_ at 240 nm [91].

For ascorbate peroxidase (APX; EC 1.11.1.11), the homogenisation step also included 2.0 mM ascorbic acid followed by centrifugation (10,000× *g*, 20 min, 4 °C). APX activity was estimated through ascorbate consumption monitored at 290 nm, using an extinction coefficient of 2.8 mM^−1^cm^−1^ [92]. All activity assays were performed at 25 °C. 

### 4.10. GC-TOF-MS Primary Metabolite Profiling Analysis

Primary metabolites were extracted from 100 mg FW fine powder of *C. glauca* branchlet tissue in 1400 μL methanol containing 60 μL of ribitol (0.2 mg mL^−1^ in water) as internal standard [93]. For more details, see Methods S3. Biological variations were controlled by analysing fatty acid methyl esters (FAMEs) internal standard markers and a quality control (QC) standard solution of 41 pure reference compounds (i.e., the most detected and abundant metabolites) throughout the analysis. Primary metabolite profiling analysis of the derivatised samples (1 µL aliquots) was performed on an Agilent 6890N gas chromatograph (Agilent Technologies, Böblingen, Germany) and a LECO Pegasus III TOF-MS running in electron ionisation (EI) mode (LECO Instrumente, Mönchengladbach, Germany) [93]. For more details, see Methods S3. GC-TOF-MS data were evaluated using the automated mass spectral deconvolution and identification system (AMDIS) software. Primary metabolites were annotated using the TagFinder software [94], matching mass spectral and retention time index to the reference collection of authenticated standard substances from the Golm Metabolome Database (GMD, http://gmd.mpimpgolm.mpg.de, last accessed on 12 December 2019) [95]. The relative abundance of primary metabolites was normalised considering the FW/dry weight (DW) ratio and the signal intensity of the internal standard (ribitol). Metadata information following the minimum reporting standard guidelines of the Metabolomics Standard Initiative (MSI) [96] can be found in Table S5.

### 4.11. LC-HRMS/MS Target Secondary Metabolite Analysis

Secondary metabolites were extracted from 100 mg FW fine powder of *C. glauca* branchlet tissue in 500 µL methanol 80% (*v/v*) containing isovitexin (4 µg mL^−1^) as internal standard. LC-HRMS/MS was performed as previously described [97]. For more details, see Methods S4. Metabolite annotation was performed following the approach described in [35]. MS/MS data interpretation was performed by matching the spectra against the METLIN MS/MS (https://metlin.scripps.edu/, last accessed on 12 December 2019) [98], NORMAN MassBank (http://massbank.eu/MassBank/, last accessed on 12 December 2019) and the MoNA (MassBank of North America) (http://mona.fiehnlab.ucdavis.edu/, last accessed on 12 December 2019) high-resolution mass spectral databases. Metadata information following the minimum reporting standard guidelines of the MSI [96] can be found in Table S5.

### 4.12. Statistical Data Analyses

Statistically significant differences between single and combined salt-heat stress treatments were determined with a two-way ANOVA, using the R software environment 3.4.3 [99]. The Benjamini–Hochberg false discovery rate (FDR) correction was used to adjust *p*-values (only for the metabolomics dataset) prior to the Tukey’s honestly significant difference (HSD) test. Fold-changes between conditions and the control for each metabolite were determined and Log_10_ transformed for heatmap plotting (R package pheatmap) [100]. Principle component analysis (PCA) and partial least squares discriminant analysis (PLS-DA) were performed on Log_10_ transformed primary metabolite relative response values using the R package “mixOmics” [101]. PCA was applied using unit variance scaling, and PLS-DA model reliability was assessed using the leave-one-out cross-validation embedded in the R package “mixOmics” [101]. Venn diagrams were obtained using the R package VennDiagram [102].

## 5. Conclusions

The present study represents a considerable advance in the field of actinorhizal plants, which was to date supported by few integrative physiological and metabolomics studies performed thus far in the model actinorhizal plant, *C. glauca*. We found that *C. glauca* could withstand naturally occurring high salt and heat stress conditions, but the harshest stress condition was demonstrated to severely compromise *C. glauca* survival. Indeed, the observed (i) stress-induced activation of plant photoprotection mechanisms (q_N_, Y_(NPQ)_, Zea, lutein, β-Carotene), (ii) increased activity of enzymatic ROS scavengers, and (iii) stress-induced activation of C and N metabolic pathways did not prevent a mesophyll limitation of photosynthesis. Faced with this situation, *C. glauca* stress resilience could not be maintained, and thus, climate change is a most urgent threat not only to stress intolerant plants but also to highly resilient plants and, as such, may ultimately represent existential stress to life on earth.

## Figures and Tables

**Table 1:** Chlorophyll a fluorescence measurements *C. glauca* branchlets under the single and combined exposure to salt (0 and 400 mM NaCl) and temperature (26, 35 and 45 °C) conditions. The parameters included: minimal fluorescence (F_o_), maximal photochemical efficiency of PSII (F_v_/F_m_) (both measured under dark-adapted conditions), the estimate of the quantum yield of non-cyclic electron transport (Y_(II)_ = Φ_e_), the quantum yield of regulated energy dissipation of PSII (Y_(NPQ)_), the quantum yield of non-regulated energy dissipation (heat and fluorescence) dissipation of PSII (Y_(NO)_), the photoprotective sustained thermal dissipation (NPQ), the non-photochemical quenching (q_N_), the photochemical quenching based on the concept of separated (q_P_) or interconnected (q_L_) antennae, the electron transport rate (ETR), the PSII photochemical efficiency under photosynthetic steady-state conditions (F_v_’/F_m_’), the predictor of the rate constant of PSII inactivation (F_s_/F_m_’), the chronic photoinhibition (PI_Chr_), the dynamic photoinhibition (PI_Dyn_) and the total photoinhibition (Total PI). Each value represents the mean ± SE of three to five independent measurements. Two-way ANOVA (*p* < 0.05) followed by Tukey’s HSD test was performed for means comparison and different letters express significant differences between [NaCl] for the same temperature (A, B) or between temperature for the same [NaCl] (c, d, e) with A and c representing the highest values.

Parameter	[NaCl]	Temperature		
		26 °C	35 °C	45 °C
**F_o_**	**0 mM**	0.216 ± 0.010 Ac	0.164 ± 0.003 Ae	0.176 ± 0.005 Ad
	**400 mM**	0.201 ± 0.005 Bc	0.170 ± 0.004 Ad	0.201 ± 0.010 Ad
**F_v_/F_m_**	**0 mM**	0.772 ± 0.008 Ac	0.818 ± 0.004 Ac	0.786 ± 0.007 Ac
	**400 mM**	0.750 ± 0.017 Ac	0.783 ± 0.009 Ac	0.489 ± 0.075 Bd
**Y_(II)_ = Ø_e_**	**0 mM**	0.533 ± 0.029 Ac	0.456 ± 0.022 Ac	0.365 ± 0.018 Ad
	**400 mM**	0.414 ± 0.034 Bc	0.376 ± 0.033 Bc	0.159 ± 0.043 Bd
**Y_(NPQ)_**	**0 mM**	0.108 ± 0.010 Bd	0.190 ± 0.019 Ad	0.254 ± 0.013 Ac
	**400 mM**	0.252 ± 0.016 Ac	0.285 ± 0.040 Ac	0.217 ± 0.067 Ac
**Y_(NO)_**	**0 mM**	0.359 ± 0.027 Ac	0.370 ± 0.026 Ac	0.381 ± 0.016 Bc
	**400 mM**	0.334 ± 0.022 Ad	0.339 ± 0.012 Ad	0.623 ± 0.106 Ac
**NPQ**	**0 mM**	0.322 ± 0.035 Bd	0.543 ± 0.061 Acd	0.670 ± 0.049 Ac
	**400 mM**	0.768 ± 0.047 Ac	0.853 ± 0.137 Ac	0.439 ± 0.181 Ad
**q_N_**	**0 mM**	0.329 ± 0.018 Bd	0.490 ± 0.031 Ac	0.529 ± 0.018 Ac
	**400 mM**	0.556 ± 0.018 Ac	0.597 ± 0.044 Ac	0.534 ± 0.069 Ac
**q_P_**	**0 mM**	0.778 ± 0.027 Ac	0.714 ± 0.017 Ac	0.590 ± 0.024 Ad
	**400 mM**	0.686 ± 0.047 Bc	0.656 ± 0.035 Bc	0.477 ± 0.045 Bd
**q_L_**	**0 mM**	0.549 ± 0.032 Ac	0.475 ± 0.019 Acd	0.356 ± 0.021 Ad
	**400 mM**	0.500 ± 0.045 Ac	0.451 ± 0.030 Acd	0.380 ± 0.029 Ad
**ETR**	**0 mM**	114.2 ± 6.3 Ac	97.7 ± 4.7 Ac	78.1 ± 3.9 Ac
	**400 mM**	88.7 ± 7.3 Bc	80.6 ± 7.1 Ac	34.1 ± 9.1 Ad
**F_v_’/F_m_’**	**0 mM**	0.679 ± 0.016 Ac	0.638 ± 0.021 Ac	0.618 ± 0.008 Ac
	**400 mM**	0.591 ± 0.019 Bc	0.571 ± 0.025 Ac	0.319 ± 0.065 Bd
**F_s_/F_m_’**	**0 mM**	0.467 ± 0.029 Bc	0.544 ± 0.022 Ac	0.635 ± 0.018 Ac
	**400 mM**	0.586 ± 0.034 Ad	0.624 ± 0.033 Acd	0.841 ± 0.043 Ac
**PI_Chr_**	**0 mM**	6.92 ± 0.77 Ac	1.55 ± 0.59 Ac	5.38 ± 0.84 Bc
	**400 mM**	8.91 ± 1.34 Ad	5.76 ± 1.44 Ad	41.2 ± 13.8 Ac
**PI_Dyn_**	**0 mM**	11.4 ± 1.3 Bd	21.7 ± 2.6 Bc	20.3 ± 1.4 Bcd
	**400 mM**	20.0 ± 1.4 Ad	25.5 ± 1.8 Ac	20.4 ± 7.4 Acd
**Total PI**	**0 mM**	18.3 ± 1.9 Bc	23.3 ± 2.5 Ac	25.7 ± 0.9 Bc
	**400 mM**	28.9 ± 2.3 Ad	31.3 ± 3.0 Ad	61.6 ± 7.8 Ac

**Table 2:** Photosynthetic pigments in *C. glauca* branchlets under the single and combined exposure to salt (0 and 400 mM NaCl) and temperature (26, 35 and 45 °C) conditions. The de-epoxidation state (DEPS) was determined as DEPS = [(Zeaxanthin (Zea) + 0.5 × Antheraxanthin (Ant))/(Violaxanthin (Viol) + Ant + Zea)]. Each value represents the mean ± SE of three to five independent measurements. Two-way ANOVA (*p* < 0.05) followed by Tukey’s HSD test was for performed for means comparison and different letters express significant differences between [NaCl] for the same temperature (A, B) or between temperature for the same [NaCl] (c, d) with A and c representing the highest values.

Pigments	[NaCl]	Temperature		
(mg g^−1^ DW)		26 °C	35 °C	45 °C
**Neoxanthin (Neo)**	**0 mM**	0.101 ± 0.006 Ac	0.121 ± 0.014 Ac	0.112 ± 0.003 Ac
	**400 mM**	0.058 ± 0.004 Bc	0.061 ± 0.006 Bc	0.069 ± 0.007 Bc
**Violaxanthin (Viol)**	**0 mM**	0.116 ± 0.008 Ac	0.120 ± 0.014 Ac	0.105 ± 0.005 Ac
	**400 mM**	0.067 ± 0.003 Bc	0.051 ± 0.009 Bc	0.042 ± 0.004 Bc
**Anteraxanthin (Ant)**	**0 mM**	0.004 ± 0.001 Ad	0.005 ± 0.000 Ad	0.013 ± 0.001 Bc
	**400 mM**	0.006 ± 0.000 Ad	0.009 ± 0.001 Ad	0.022 ± 0.002 Ac
**Zeaxanthin (Zea)**	**0 mM**	0.000 ± 0.000 Bd	0.002 ± 0.001 Bd	0.011 ± 0.003 Bc
	**400 mM**	0.003 ± 0.000 Ad	0.015 ± 0.005 Ad	0.035 ± 0.007 Ac
**Viol+Ant+Zea**	**0 mM**	0.120 ± 0.008 Ac	0.126 ± 0.012 Ac	0.129 ± 0.002 Ac
	**400 mM**	0.075 ± 0.003 Bc	0.075 ± 0.004 Bc	0.099 ± 0.011 Bc
**DEPS**	**0 mM**	0.018 ± 0.002 Ac	0.046 ± 0.021 Bc	0.139 ± 0.023 Bc
	**400 mM**	0.070 ± 0.005 Ad	0.282 ± 0.077 Ad	0.442 ± 0.034 Ac
**Lutein**	**0 mM**	0.257 ± 0.018 Ad	0.338 ± 0.037 Acd	0.367 ± 0.008 Ac
	**400 mM**	0.174 ± 0.011 Bd	0.196 ± 0.015 Bcd	0.245 ± 0.023 Bc
**α-Carotene**	**0 mM**	0.019 ± 0.002 Ad	0.054 ± 0.001 Ac	0.050 ± 0.004 Ac
	**400 mM**	0.032 ± 0.005 Ad	0.050 ± 0.010 Ac	0.055 ± 0.005 Ac
**β-Carotene**	**0 mM**	0.007 ± 0.001 Ad	0.020 ± 0.002 Ac	0.017 ± 0.001 Ac
	**400 mM**	0.007 ± 0.001 Ac	0.011 ± 0.002 Bc	0.013 ± 0.002 Ac

**Table 3:** Total fatty acids (TFA, mg g^−1^ DW), fatty acid composition (mol%) and unsaturation (DBI) of *C. glauca* branchlets under the single and combined exposure to salt (0 and 400 mM NaCl) and temperature (26, 35 and 45 °C) conditions. C18:3-linolenic acid. C18:2-linoleic acid. C16:0-palmitic acid. C18:1-oleic acid. C18:0-stearic acid. C16:1-hexadecenoic acid and less representative (Less Rep) fatty acids (i.e., the sum of less abundant FAs, namely C15:0–pentadecanoic acid, C14:0–myristic acid and C14:1–myristoleic acid). DBI = [(%monoenes + 2x%dienes + 3x%trienes)/%saturated FAs]. Each value represents the mean ± SE of three to five independent measurements. Two-way ANOVA (*p* < 0.05) followed by Tukey’s HSD test was performed for means comparison, and different letters express significant differences between [NaCl] for the same temperature (A, B) or between temperature for the same [NaCl] (c, d) with A and c representing the highest values.

Parameter	[NaCl]	Temperature (°C)		
		26 °C	35 °C	45 °C
**TFA (mg g^−1^ DW)**	**0 mM**	13.65 ± 1.50 Ac	12.80 ± 1.17 Ac	12.26 ± 0.34 Ac
	**400 mM**	8.10 ± 1.40 Bc	7.94 ± 0.53 Bc	6.80 ± 0.65 Bc
**C18:3 (mol%)**	**0 mM**	41.52 ± 0.27 Ac	35.75 ± 1.90 Ac	37.05 ± 0.41 Ac
	**400 mM**	33.82 ± 2.44 Bc	34.72 ± 1.01 Ac	24.53 ± 2.43 Bd
**C18:2 (mol%)**	**0 mM**	19.51 ± 0.33 Bd	22.50 ± 1.03 Ac	21.93 ± 0.60 Bcd
	**400 mM**	22.94 ± 0.55 Ac	24.15 ± 0.44 Ac	23.87 ± 1.35 Ac
**C18:1 (mol%)**	**0 mM**	5.73 ± 0.08 Ad	7.35 ± 0.26 Acd	7.66 ± 0.24 Bc
	**400 mM**	6.06 ± 0.76 Ad	6.28 ± 0.83 Acd	8.16 ± 0.56 Ac
**C18:0 (mol%)**	**0 mM**	2.65 ± 0.21 Ac	2.64 ± 0.24 Ac	2.00 ± 0.19 Ac
	**400 mM**	2.92 ± 0.27 Ac	2.89 ± 0.30 Ac	3.25 ± 0.42 Ac
**C16:1 (mol%)**	**0 mM**	3.68 ± 0.23 Ac	3.39 ± 0.40 Ac	3.92 ± 0.12 Ac
	**400 mM**	3.76 ± 0.23 Ac	3.17 ± 0.03 Ac	3.27 ± 0.28 Ac
**C16:0 (mol%)**	**0 mM**	25.67 ± 0.68 Ac	27.12 ± 2.35 Ac	26.30 ± 0.80 Bc
	**400 mM**	28.36 ± 1.60 Acd	26.88 ± 1.18 Ad	34.67 ± 2.90 Ac
**Less Rep (mol%)**	**0 mM**	1.13 ± 0.07 Ac	1.06 ± 0.14 Ac	0.95 ± 0.04 Ac
	**400 mM**	1.13 ± 0.18 Ac	1.02 ± 0.07 Ac	1.01 ± 0.14 Ac
**DBI**	**0 mM**	6.47 ± 0.22 Ac	5.94 ± 0.76 Ac	6.10 ± 0.25 Ac
	**400 mM**	5.25 ± 0.48 Ac	5.67 ± 0.31 Ac	3.77 ± 0.65 Bd

## Data Availability

The data presented in this study are available in article and Supplementary Materials.

## Supplementary Materials

The following are available online at https://www.mdpi.com/article/10.3390/metabo11090593/s1, Figure S1 Diagram of the experi-mental design, Figure S2 LC-HRMS/MS secondary metabolite analysis, Figure S3 principal component analysis (PCA) score plot of the secondary metabolite profiles, Figure S4 partial least square discriminant analysis (PLS-DA) of the secondary metabolite profiles, Table S1 photosyn-thetic pigments—HPLC chromatograms, Table S2 GC-TOF-MS primary metabolite profiling of *C. glauca* branchlets under the single and combined exposure to salt (0 and 400 mM NaCl) and temperature (26, 35 and 45 °C) conditions, Table S3 annotation of the secondary metabolites de-tected by LC-HRMS/MS in *C. glauca* branchlets, Table S4 LC-HRMS/MS secondary metabolite analysis of *C. glauca* branchlets under the single and combined exposure to salt (0 and 400 mM NaCl) and temperature (26, 35 and 45 °C) conditions, Table S5 metabolomics metadata infor-mation, Methods S1 detailed description of the chlorophyll a parameters evaluated under pho-tosynthetic steady-state conditions, Methods S2 lipid analyses, Methods S3 primary metabolite extraction, derivatisation and GC-TOF-MS analysis, Methods S4 secondary metabolite extraction and LC-HRMS analysis [103,104,105,106].
