# Supplementary material for: Will Casuarina glauca Stress Resilience Be Maintained in the Face of Climate Change?
Source: Metabolites. 2021 Sep 2;11(9):593. doi: 10.3390/metabo11090593 (PMC8467279; doi:10.3390/metabo11090593)
Supplement: Supplementary file 1 [file metabolites-11-00593-s001.zip › metabolites-1356915-supplementary/Supplementary Information/Supplementary Information Jorge et al 2021.pdf]

Supplementary Information

Article title: Will Casuarina glauca stress resilience be maintained in the face of climate change?

Authors: Tiago F. Jorge, José C. Ramalho, Saleh Alseekh, Isabel P. Pais, António E. Leitão, Ana P. Rodrigues, Paula Scotti-Campos, Ana I. Ribeiro-Barros, Alisdair R. Fernie and Carla António
The following Supporting Information is available for this article:

Table S1 Photosynthetic pigments – HPLC chromatograms

Figure S1 Diagram of the experimental design.

Figure S2 LC-HRMS/MS secondary metabolite analysis.

Figure S3 Principal component analysis (PCA) score plot of the secondary metabolite profiles.

Figure S4 Partial least square discriminant analysis (PLS-DA) of the secondary metabolite profiles.

Table S2 GC-TOF-MS primary metabolite profiling of *C. glauca* branchlets under the single and combined exposure to salt (0 and 400 mM NaCl) and temperature (26, 35 and 45 ºC) conditions.

Table S3 Annotation of the secondary metabolites detected by LC-HRMS/MS in *C. glauca* branchlets.

Table S4 LC-HRMS/MS secondary metabolite analysis of *C. glauca* branchlets under the single and combined exposure to salt (0 and 400 mM NaCl) and temperature (26, 35 and 45 ºC) conditions.

Table S5 Metabolomics metadata information.

Methods S1 Detailed description of the chlorophyll a parameters evaluated under photosynthetic steady-state conditions

Methods S2 Lipid analyses

Methods S3 Primary metabolite extraction, derivatisation, and GC-TOF-MS analysis

Methods S4 Secondary metabolite extraction, and LC-HRMS analysis

File S1 Photosynthetic pigments – HPLC chromatograms

Figure S1 Diagram of the experimental design. Salt-heat stress experiment and subsequent physiological and MS-based metabolomics analyses. Three to five *C. glauca* plants were used per independent treatment.

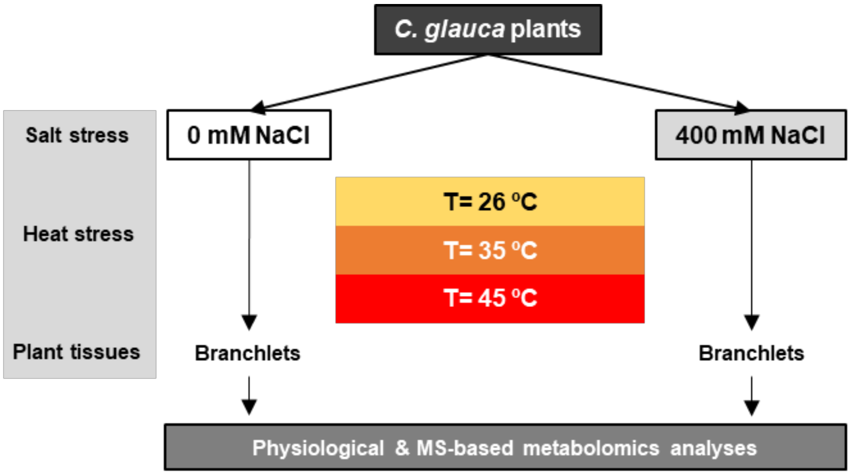

Figure S2 LC-HRMS/MS secondary metabolite analysis. Heatmap showing metabolite responses in *C. glauca* branchlets under the single and combined exposure to salt (0 and 400 mM NaCl) and temperature (26, 35 and 45 ºC) conditions. Relative values are normalised to the internal standard (isovitexin) and dry weight (DW) of the samples. False-colour imaging was performed on Log_{10}-transformed LC-HRMS/MS data.

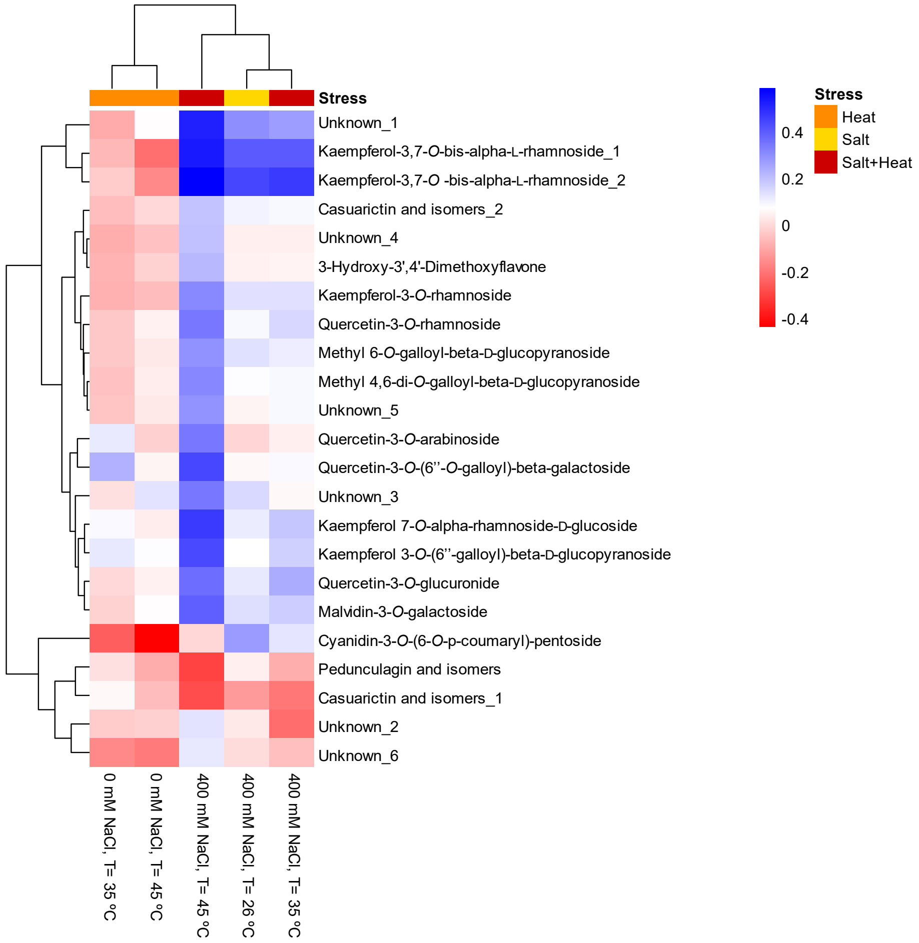

Figure S3 Principal component analysis (PCA) score plot of the secondary metabolite profiles. Score plot of the secondary metabolite profiles in the branchlets of *C. glauca* plants under the single and combined exposure to salt (0 and 400 mM NaCl) and temperature (26, 35 and 45 ºC) conditions.

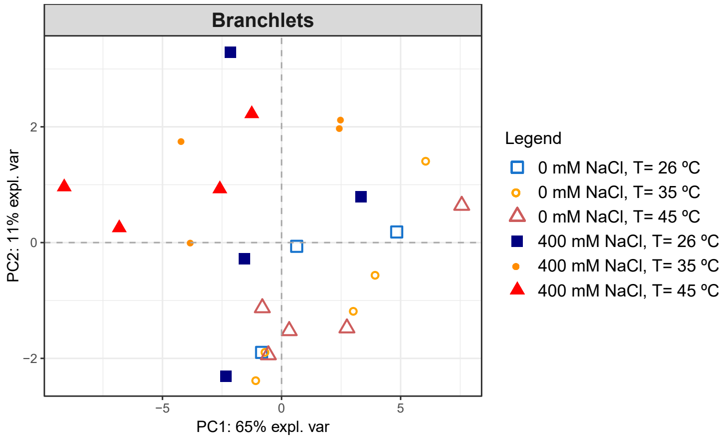

Figure S4 Partial least square discriminant analysis (PLS-DA) of the secondary metabolite profiles. PLS-DA score and contribution plot in the branchlets of *C. glauca* plants under the single and combined exposure to salt (0 and 400 mM NaCl) and temperature (26, 35 and 45 ºC) conditions. The bar length in the contribution plots represents the loading weights of each metabolite in component 1. The colour indicates the stress condition in which the metabolite has a maximal importance (based on the median).

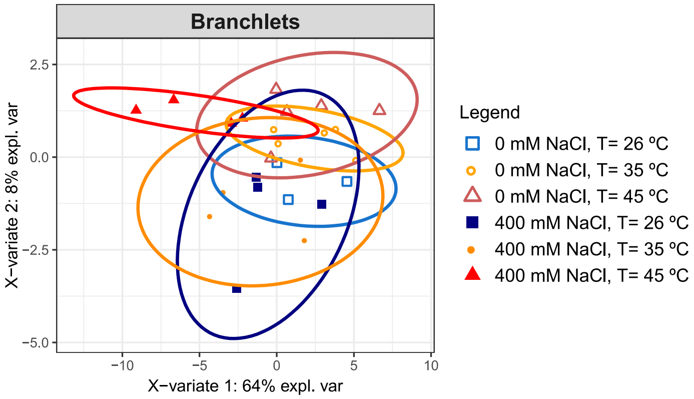

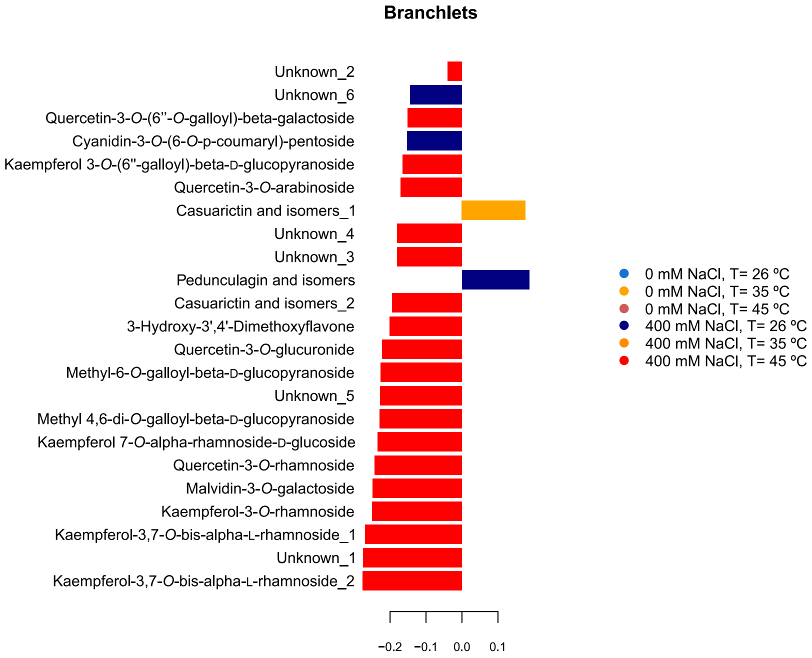

Methods S1 Detailed description of the chlorophyll a parameters evaluated under photosynthetic steady-state conditions

A set of parameters was evaluated under photosynthetic steady-state conditions, using *ca.* 510 µmol m^{-2} s^{-1} of actinic light and superimposed saturating flashes. This included q_P, q_L, NPQ, $Y_{(II)}$, $Y_{(NPQ)}$, $Y_{(NO)}$ and F_v'/F_m'. F_o' needed for q_P determination was obtained immediately after switching off actinic light, before the first fast phase of fluorescence relaxation kinetics in the dark. The F_v'/F_m' represents the estimated actual PSII photochemical efficiency of energy conversion under light; q_P and q_L denote the proportion of energy trapped by PSII open centers and driven to photochemical events, based on the concept of separated (q_P) or interconnected (q_L) PSII antennae; NPQ is the non-photochemical quenching, representing the photoprotective sustained thermal energy dissipation. Estimates of the quantum yields of photosynthetic non-cyclic electron transport ($Y_{(II)} = \Phi_e$), of regulated energy dissipation of PSII ($Y_{(NPQ)}$), and of non-regulated energy (heat and fluorescence) dissipation of PSII ($Y_{(NO)}$) were also performed [41, 43]. The PSII photoinhibition indexes were determined as described in [103] Werner and included chronic photoinhibition (PI_{Chr}), dynamic photoinhibition (PI_{Dyn}) and total photoinhibition (Total PI= PI_{Chr} + PI_{Dyn}). The chronic photoinhibition (PI_{Chr}) represents the long-term sustainable decrease in F_v/F_m and was calculated as the percentage reduction in predawn F_v/F_m (PD) relative to the maximal F_v/F_m (Max) obtained during the entire experiment. The dynamic photoinhibition (PI_{Dyn}) represents the decline in F_v/F_m that is fully reversible overnight and was measured as the percent reduction of midday F_v'/F_m' (MD) relative to the predawn F_v/F_m (PD) at each temperature in relation to the maximal F_v/F_m (Max) from the entire experiment.

Methods S2 Lipid analyses

Lipid analysis was performed as previously described in Campos *et al,.* (2003). Separation was carried out on a DB-Wax capillary column (J & W Scientific, 0.25 mm i.d. x 30 m, 0.25 µm; Agilent, CA, USA). Column temperature was programmed to rise from 80 ºC to 200 ºC at a rate of 12 ºC min^{-1}, after 2 min at the initial temperature. Injector and detector temperatures were 200 ºC and

250 ºC, respectively. Hydrogen was used as carrier gas at a flow rate of 1 mL min^{-1}, and with a split ratio of 1:100. FAs were identified by comparison with known standard compounds (Sigma, USA). The value of the total fatty acids (TFA) was calculated through the sum of individual FAs.

Methods S3 Primary metabolite extraction, derivatisation, and GC-TOF-MS analysis

Primary metabolites were extracted following a previously described method [93]. Aliquots of 100 mg fresh weight (FW) of finely homogenised plant tissue were dissolved in 1400 µL of methanol containing 60 µL of ribitol (0.2 mg mL^{-1} in water) as the internal standard. The mixture was incubated at 70 ºC on a shaker and then centrifuged at 25 ºC, 11000 *g*, for 10 min. The supernatant was transferred to a new tube and mixed with 750 µL of chloroform followed by 1500 µL of water. The mixture was then centrifuged for 15 min at 2200 *g* and an aliquot of 150 µL of the upper polar phase was dried in a vacuum concentrator (LabConco Centrivap 78100 Series, USA) for at least 3 h at 30 ºC. The dried samples were dissolved and derivatised using a two-step procedure involving methoxyamination and trimethylsilylation as previously described [93]. Biological variations were controlled by analysing fatty acid methyl esters (FAMES) internal standard markers and a quality control (QC) standard solution of 41 pure reference compounds (i.e. the most detected and abundant metabolites) throughout the analysis.

Metabolite profiling analysis of the derivatised samples (1 µL aliquots) was performed on an Agilent 6890N gas chromatograph (Agilent Technologies, Böblingen, Germany), and a LECO Pegasus III TOF-MS running in electron ionisation (EI) mode (LECO Instrumente, Mönchengladbach, Germany). The chromatographic separation was performed on a VF-5MS column (Varian Inc., 30 m length, 0.25 mm inner diameter, and 0.25 µm film thickness). The temperature program was set as follows: isothermal for 2 min at 85 ºC, followed by a 15 ºC per min ramp to 360 ºC, and hold at this temperature for 6 min. The injector and transfer line temperatures were set to 230 ºC and 250 ºC, respectively, and the injection was performed both in the splitless and split (1:30) mode with helium carrier gas flow set to 2 mL min^{-1}. After a solvent delay of 180 seconds, mass spectra were scanned from m/z 70-600 with acquisition rate of 20 spectra s^{-1} and a detector voltage between 1700 and 1850 V.

Methods S4 Secondary metabolite extraction, and LC-HRMS analysis

Secondary metabolite extraction was performed according to a previously described method [97]. Briefly, 190 mg fresh weight (FW) of plant tissue powder were extracted in 500 µL of methanol 80 % (v/v) containing isovitexin (4 µg mL^{-1}) as the internal standard. The mixture was centrifuged for 10 minutes at 12000 *g*. The supernatant was transferred to a new tube (1.5 mL) and it was centrifuged again for 10 min at 12000 *g*. The remaining supernatant was transferred to a new tube (1.5 mL) and stored at 4 ºC before analysis. Secondary metabolite profiling analysis was performed on a Waters Acquity UPLC liquid chromatograph (Waters, USA) coupled to a Thermo Q Exactive Orbitrap mass spectrometer (ThermoFisher, USA). UPLC analysis were performed on a reverse-phase C18 HSS T3 column (1.8 µm, 100 mm x 2.1 mm i.d., Waters, USA) at 40 ºC using a binary mobile phase composed of (A) water containing 0.1 % formic acid and (B) acetonitrile containing 0.1 % formic acid. The flow rate was set to 400 µL min^{-1} sample injection was 5 µL. The gradient elution program was as follows: 0-1 min 99 % A + 1 % B; 1-11 min 99 % A + 1 % B to 60% A + 40 % B; 11-13 min 60 % A + 40 % B to 30 % A to 70 % B; 13-15 min 30 % A + 70 % B to 1 % A + 99% B and maintained for 1 min; 16-17 min 1 % A + 99 % B to 99 % A + 1 % B, and maintained for 3 min (20 min total run time). LC-HRMS instrument parameters were performed as follows: ion source voltage 3.5 kV, capillary temperature 320 ºC, sheath and auxiliary gases 60 and 20 (arbitrary units), respectively. Mass spectra were acquired over the scan range m/z 100-1500 and using a mass resolution of 70000. All Ion Fragmentation (AIF) MS/MS data acquisition was set as follows: ion source voltage 3.5 kV, capillary temperature 320 ºC, sheath and auxiliary gases 60 and 20 (arbitrary units), respectively, and higher energy collisional dissociation (HCD) of 30 %. Mass spectra were acquired over the scan range m/z 100-1000 and using a mass resolution of 17500.
