# Supplementary material for: Will Casuarina glauca Stress Resilience Be Maintained in the Face of Climate Change?
Source: Metabolites. 2021 Sep 2;11(9):593. doi: 10.3390/metabo11090593 (PMC8467279; doi:10.3390/metabo11090593)
Supplement: Supplementary file 1 [file metabolites-11-00593-s001.zip › metabolites-1356915-supplementary/Supplementary Information/Table S1.pdf]

Supplementary Information Table S1

Article title: Will Casuarina glauca stress resilience be maintained in the face of climate change?

Authors: Tiago F. Jorge, José C. Ramalho, Saleh Alseekh, Isabel P. Pais, António E. Leitão, Ana P. Rodrigues, Paula Scotti-Campos, Ana I. Ribeiro-Barros, Alisdair R. Fernie and Carla António

Table S1 Photosynthetic pigments – HPLC chromatograms

0 mM NaCl, T= 26ºC

1

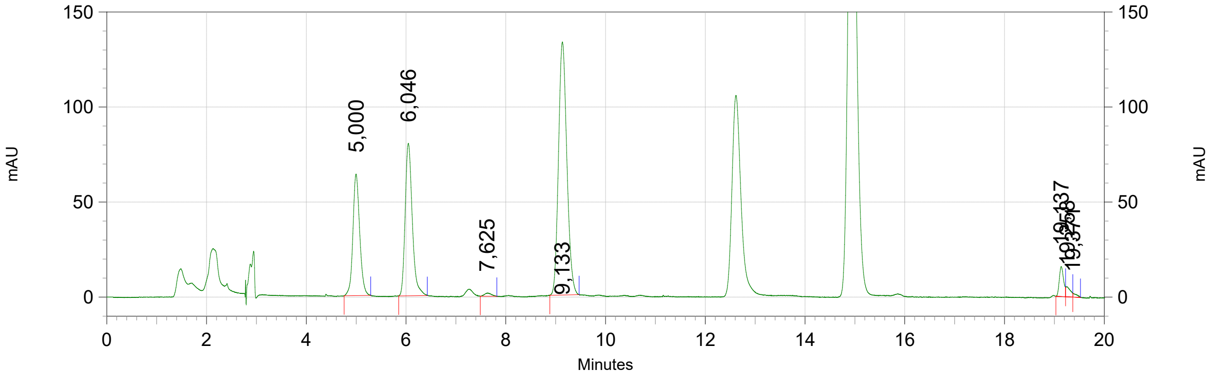

Det 168-440nm

Name	Retention Time	Area	Area Percent
Neoxanthin	5.000	604162	19.477
Violaxanthin	6.046	791475	25.515
Anteraxanthin	7.625	16408	0.529
Lutein	9.133	1550615	49.988
Zeaxanthin		0	
Alfa carotene	19.137	94505	3.047
Beta carotene	19.258	34430	1.110

2

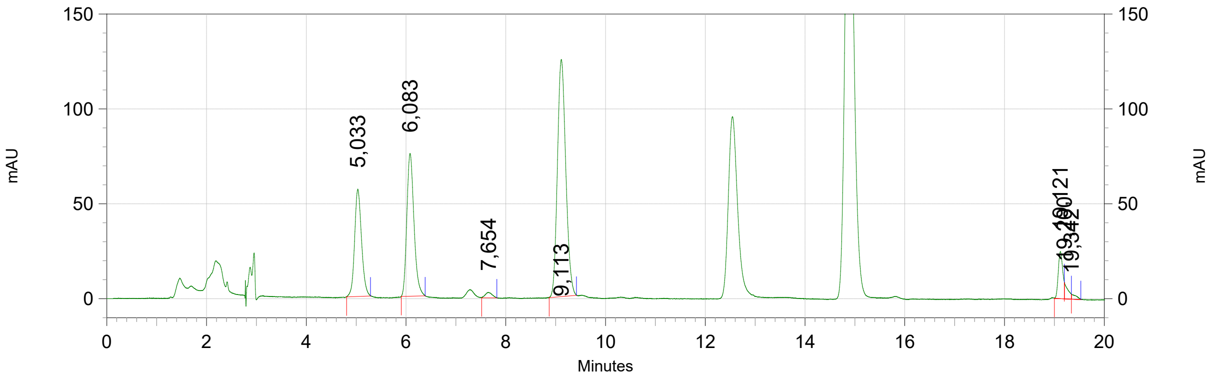

Det 168-440nm

Name	Retention Time	Area	Area Percent
Neoxanthin	5.033	543465	18.261
Violaxanthin	6.083	745318	25.043
Anteraxanthin	7.654	26086	0.877
Lutein	9.113	1452883	48.817
Zeaxanthin		0	
Alfa carotene	19.121	151389	5.087
Beta carotene	19.200	41480	1.394

3

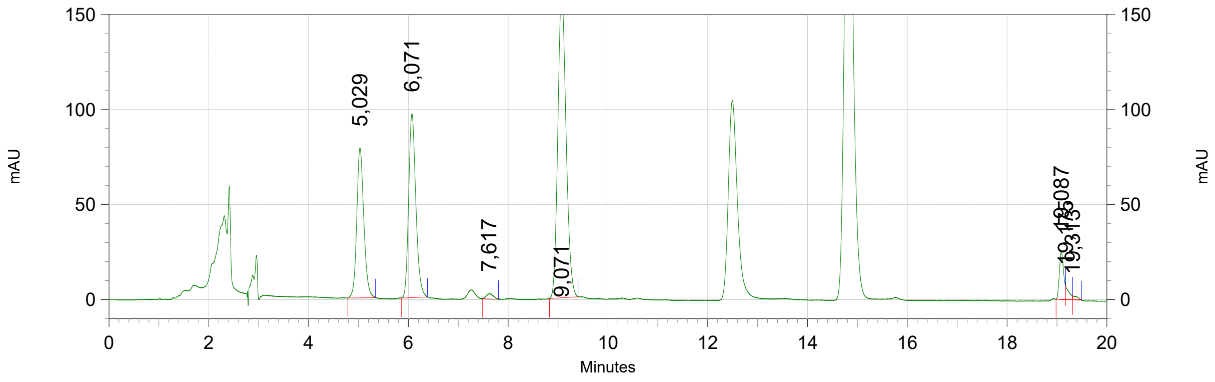

Det 168-440nm			
Name	Retention Time	Area	Area Percent
Neoxanthin	5.029	774619	20.366
Violaxanthin	6.071	962543	25.307
Anteraxanthin	7.617	27246	0.716
Lutein	9.071	1845081	48.510
Zeaxanthin		0	
Alfa carotene	19.087	147845	3.887
Beta carotene	19.175	32756	0.861

400 mM NaCl, T= 26ºC

5

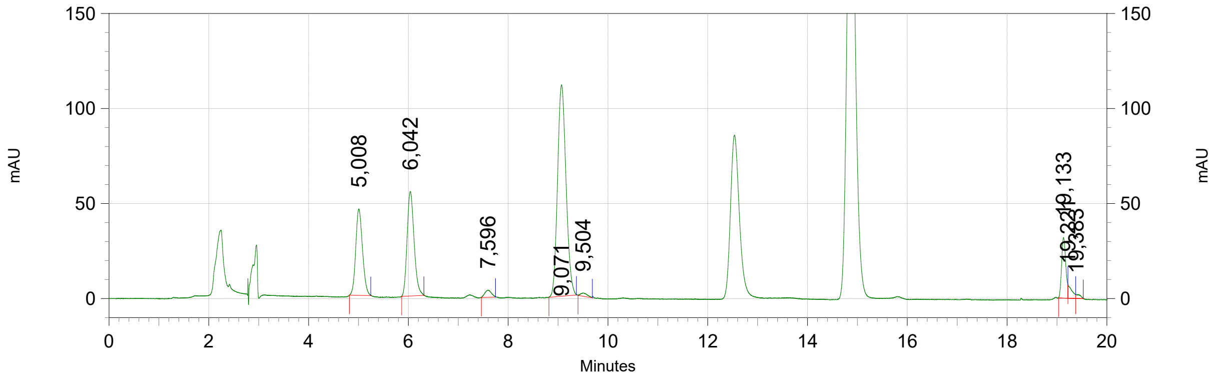

Det 168-440nm			
Name	Retention Time	Area	Area Percent
Neoxanthin	5.008	426485	16.744
Violaxanthin	6.042	532038	20.888
Anteraxanthin	7.596	33049	1.297
Lutein	9.071	1310319	51.443
Zeaxanthin	9.504	14766	0.580
Alfa carotene	19.133	181975	7.144
Beta carotene	19.221	37059	1.455

6

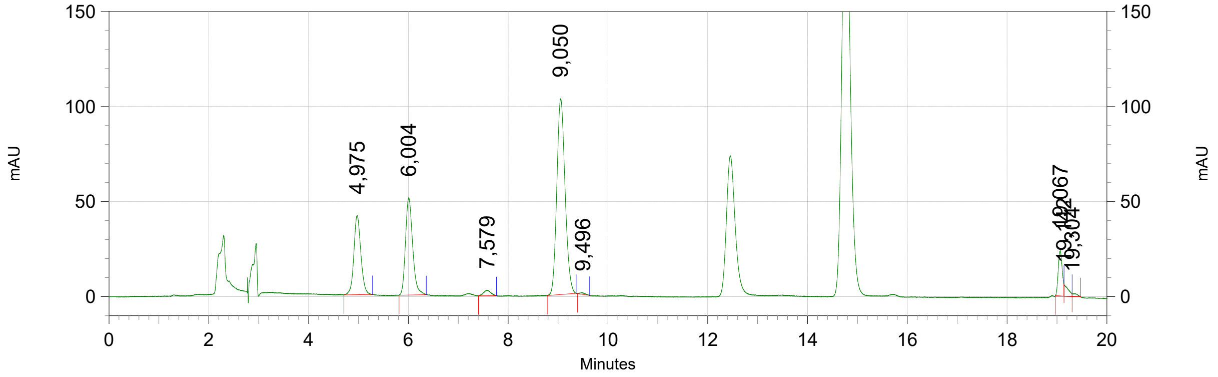

Det 168-440nm

Name	Retention Time	Area	Area Percent
Neoxanthin	4.975	405382	17.550
Violaxanthin	6.004	508922	22.032
Anteraxanthin	7.579	28155	1.219
Lutein	9.050	1182237	51.181
Zeaxanthin	9.496	6162	0.267
Alfa carotene	19.067	133180	5.766
Beta carotene	19.142	35742	1.547

8

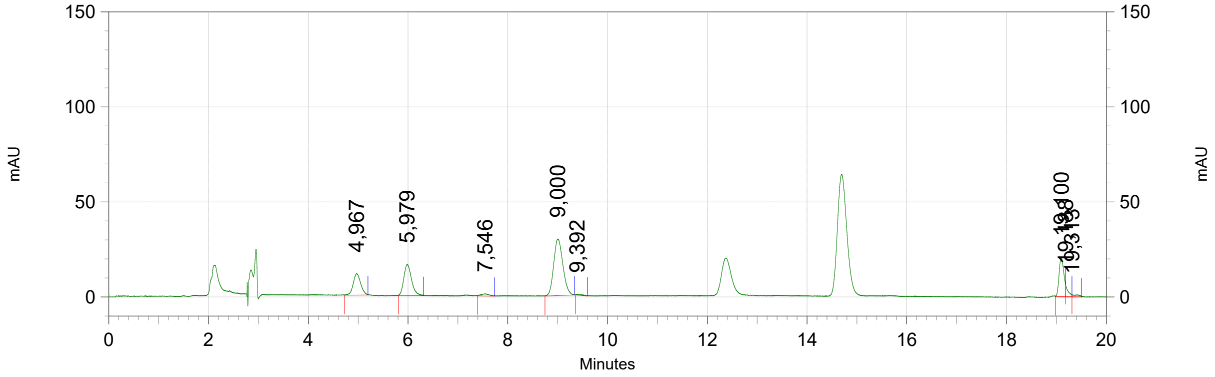

Det 168-440nm

Name	Retention Time	Area	Area Percent
Neoxanthin	4.967	116454	13.844
Violaxanthin	5.979	173284	20.599
Anteraxanthin	7.546	10742	1.277
Lutein	9.000	376612	44.770
Zeaxanthin	9.392	2846	0.338
Alfa carotene	19.100	134511	15.990
Beta carotene	19.188	19082	2.268

0 mM NaCl, T= 35ºC

10

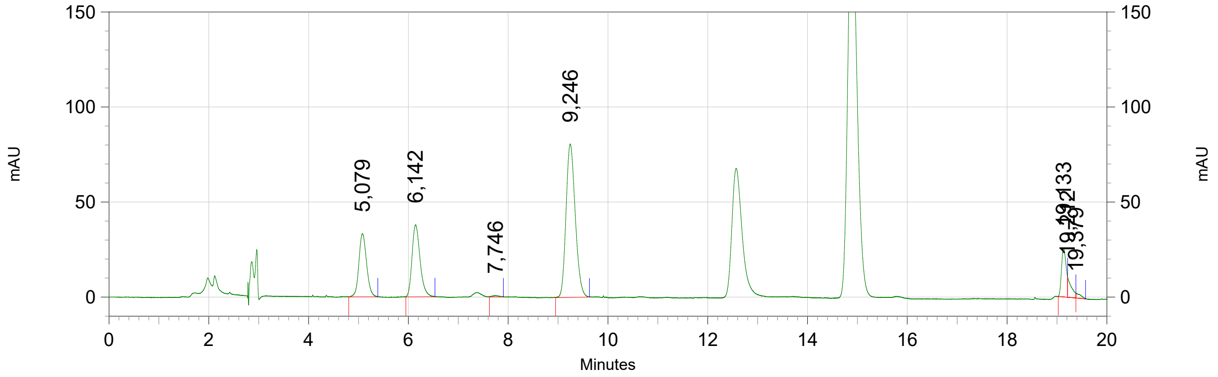

Det 168-440nm

Name	Retention Time	Area	Area Percent
Neoxanthin	5.079	356713	17.307
Violaxanthin	6.142	425094	20.625
Anteraxanthin	7.746	6816	0.331
Lutein	9.246	1036667	50.297
Zeaxanthin		0	
Alfa carotene	19.133	162898	7.904
Beta carotene	19.212	56432	2.738

16

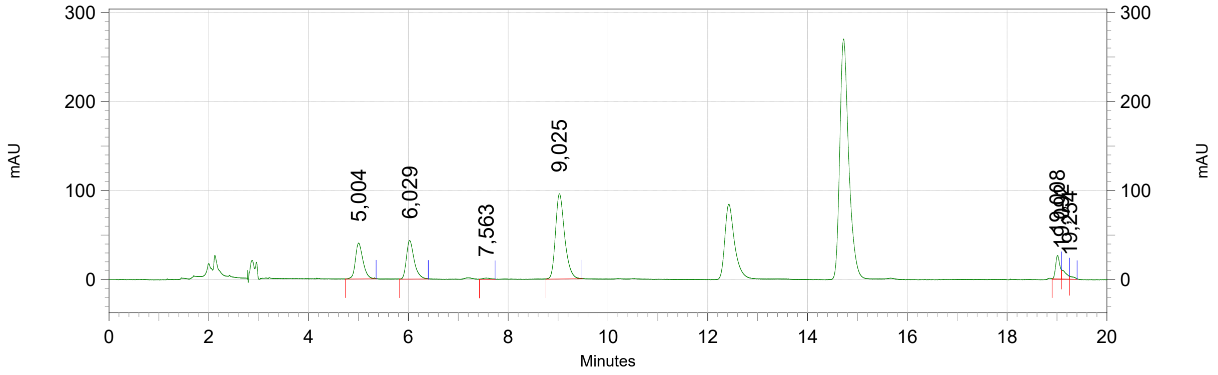

Det 168-440nm

Name	Retention Time	Area	Area Percent
Neoxanthin	5.004	424455	18.162
Violaxanthin	6.029	463677	19.841
Anteraxanthin	7.563	11726	0.502
Lutein	9.025	1207669	51.676
Zeaxanthin		0	
Alfa carotene	19.008	152266	6.515
Beta carotene	19.092	62253	2.664

17

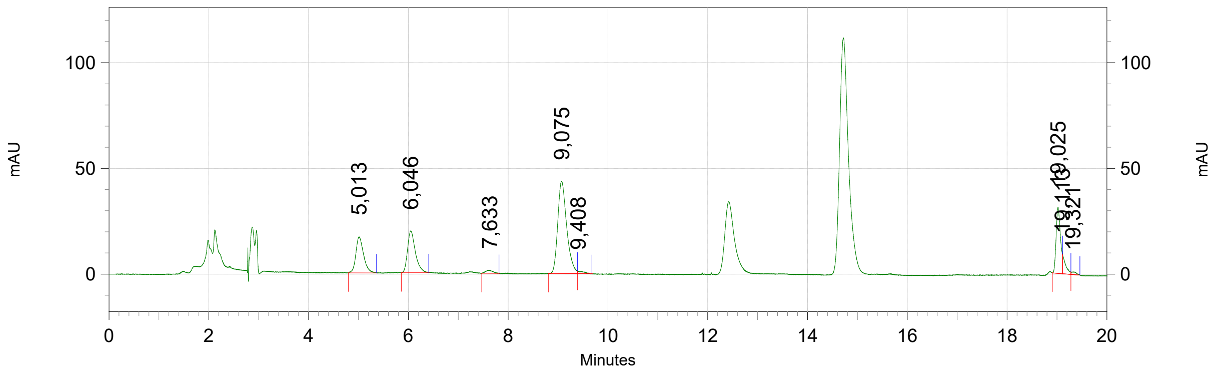

Det 168-440nm

Name	Retention Time	Area	Area Percent
Neoxanthin	5.013	182530	15.258
Violaxanthin	6.046	215234	17.991
Anteraxanthin	7.633	15216	1.272
Lutein	9.075	540266	45.161
Zeaxanthin	9.408	9061	0.757
Alfa carotene	19.025	187214	15.649
Beta carotene	19.113	37876	3.166

18

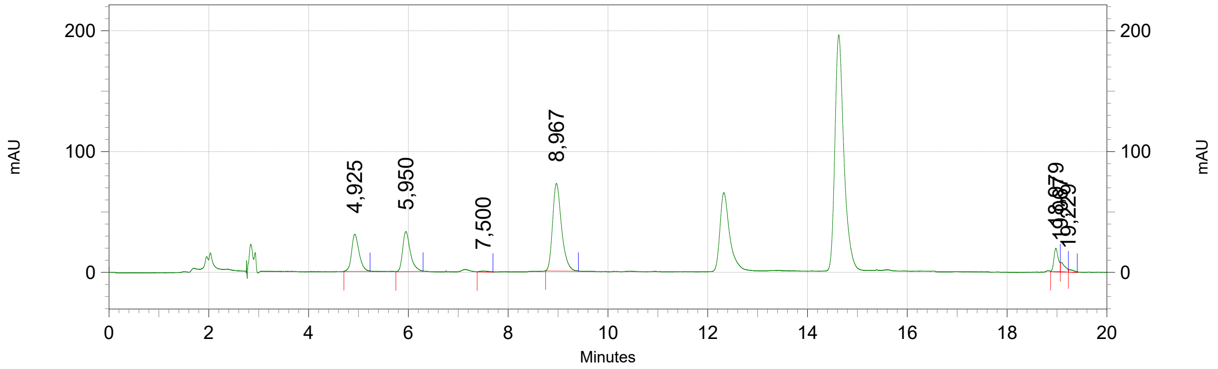

Det 168-440nm

Name	Retention Time	Area	Area Percent
Neoxanthin	4.925	319474	18.207
Violaxanthin	5.950	352917	20.113
Anteraxanthin	7.500	8272	0.471
Lutein	8.967	899786	51.280
Zeaxanthin		0	
Alfa carotene	18.979	115866	6.603
Beta carotene	19.067	46919	2.674

400 mM NaCl, T= 35ºC

4

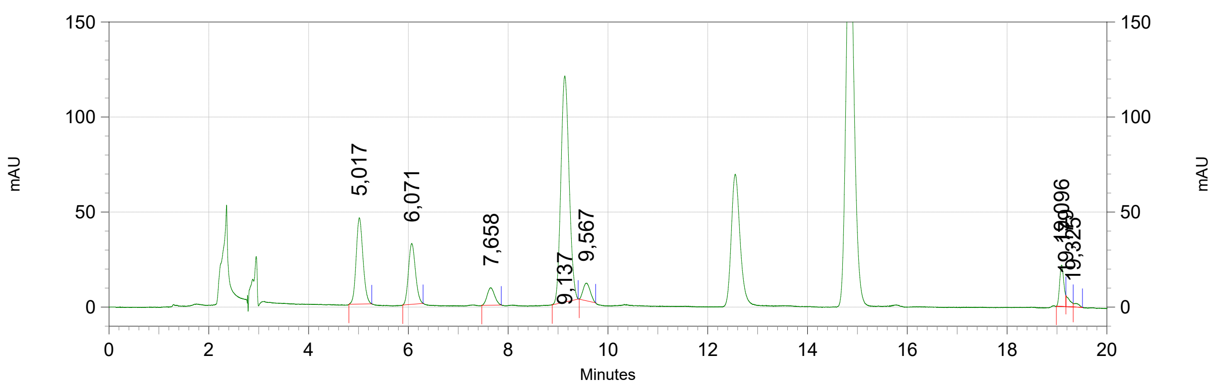

Det 168-440nm			
Name	Retention Time	Area	Area Percent
Neoxanthin	5.017	431137	17.488
Violaxanthin	6.071	309627	12.559
Anteraxanthin	7.658	95564	3.876
Lutein	9.137	1361881	55.241
Zeaxanthin	9.567	92365	3.747
Alfa carotene	19.096	128814	5.225
Beta carotene	19.179	31543	1.279

9

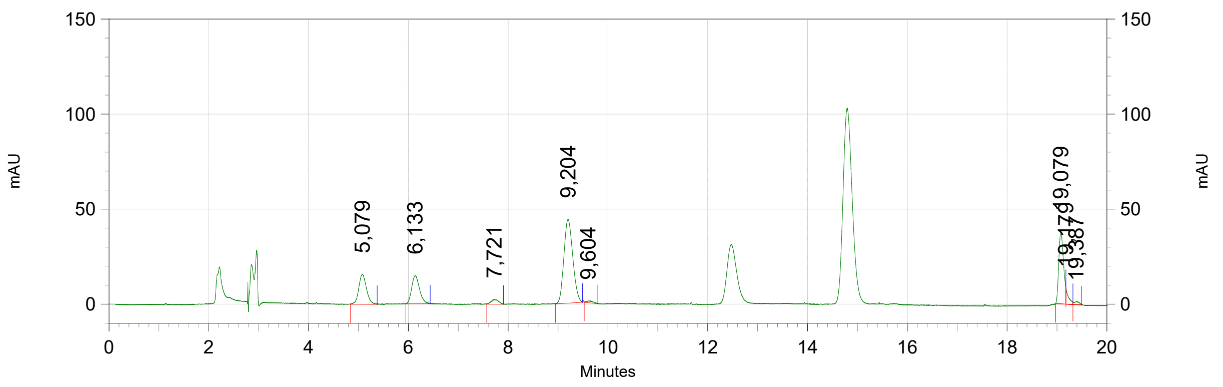

Det 168-440nm			
Name	Retention Time	Area	Area Percent
Neoxanthin	5.079	167498	14.115
Violaxanthin	6.133	160013	13.485
Anteraxanthin	7.721	24203	2.040
Lutein	9.204	542368	45.707
Zeaxanthin	9.604	7774	0.655
Alfa carotene	19.079	247223	20.834
Beta carotene	19.179	27704	2.335

13

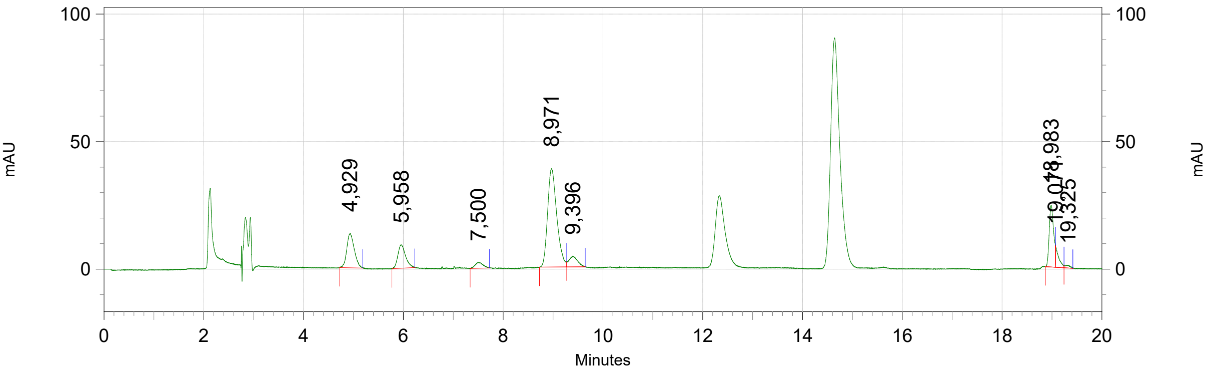

Det 168-440nm

Name	Retention Time	Area	Area Percent
Neoxanthin	4.929	140852	14.092
Violaxanthin	5.958	98812	9.886
Anteraxanthin	7.500	25301	2.531
Lutein	8.971	493183	49.341
Zeaxanthin	9.396	49692	4.971
Alfa carotene	18.983	148306	14.837
Beta carotene	19.071	35789	3.581

20

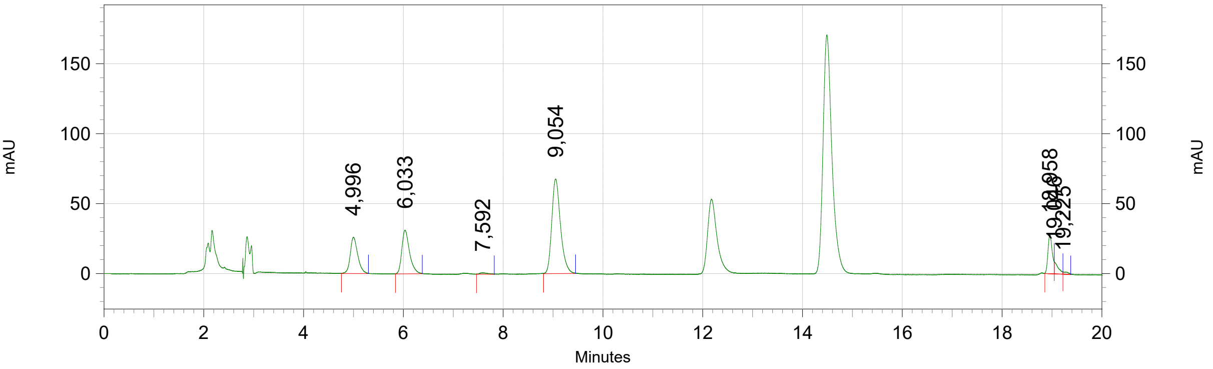

Det 168-440nm

Name	Retention Time	Area	Area Percent
Neoxanthin	4.996	269239	16.325
Violaxanthin	6.033	333525	20.223
Anteraxanthin	7.592	10119	0.614
Lutein	9.054	817914	49.595
Zeaxanthin		0	
Alfa carotene	18.958	164407	9.969
Beta carotene	19.046	44856	2.720

0 mM NaCl, T= 45ºC

11

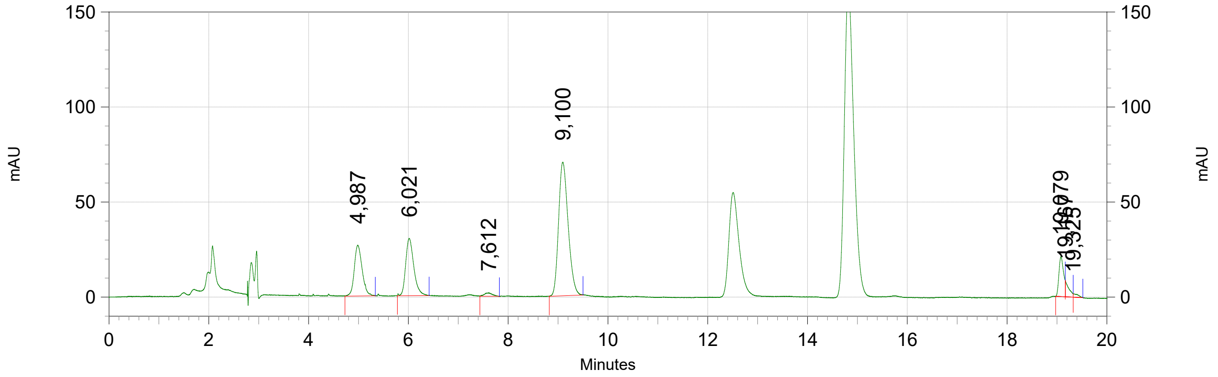

Det 168-440nm

Name	Retention Time	Area	Area Percent
Neoxanthin	4.987	293062	16.480
Violaxanthin	6.021	342026	19.233
Anteraxanthin	7.612	19812	1.114
Lutein	9.100	934275	52.537
Zeaxanthin		0	
Alfa carotene	19.079	134398	7.558
Beta carotene	19.167	42422	2.386

14

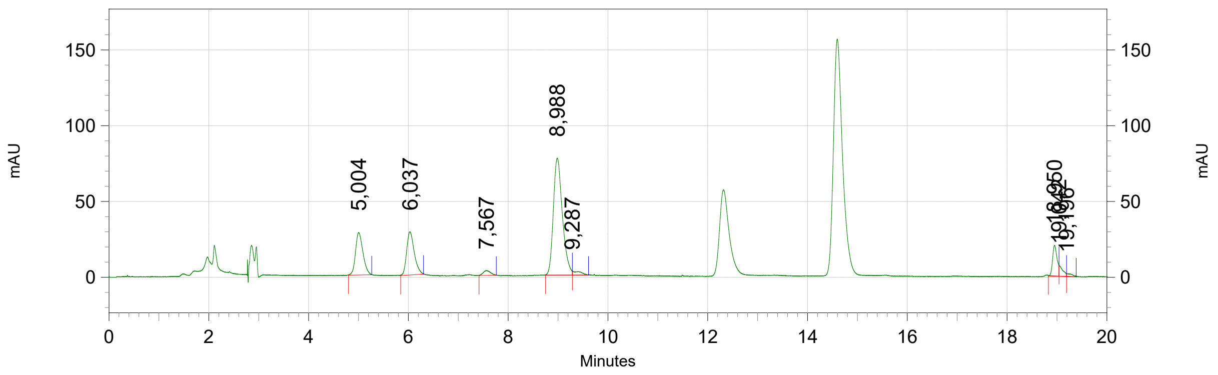

Det 168-440nm

Name	Retention Time	Area	Area Percent
Neoxanthin	5.004	287209	16.126
Violaxanthin	6.037	299474	16.815
Anteraxanthin	7.567	30798	1.729
Lutein	8.988	966063	54.242
Zeaxanthin	9.287	26709	1.500
Alfa carotene	18.950	123449	6.931
Beta carotene	19.042	35550	1.996

19

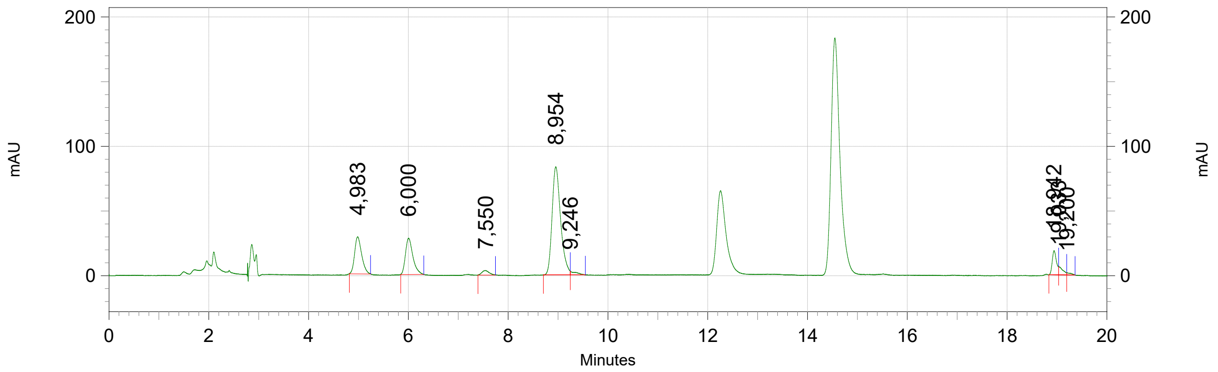

Det 168-440nm

Name	Retention Time	Area	Area Percent
Neoxanthin	4.983	280820	15.799
Violaxanthin	6.000	290722	16.356
Anteraxanthin	7.550	35409	1.992
Lutein	8.954	990184	55.707
Zeaxanthin	9.246	22015	1.239
Alfa carotene	18.942	110254	6.203
Beta carotene	19.033	37784	2.126

22

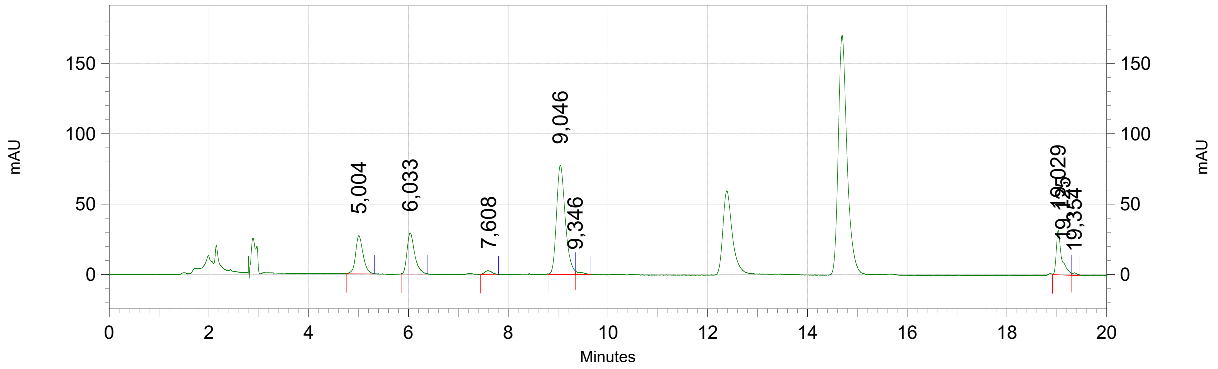

Det 168-440nm

Name	Retention Time	Area	Area Percent
Neoxanthin	5.004	282059	15.514
Violaxanthin	6.033	309679	17.033
Anteraxanthin	7.608	27690	1.523
Lutein	9.046	940540	51.732
Zeaxanthin	9.346	18281	1.006
Alfa carotene	19.029	186486	10.257
Beta carotene	19.125	44938	2.472

400 mM NaCl, T= 45ºC

7

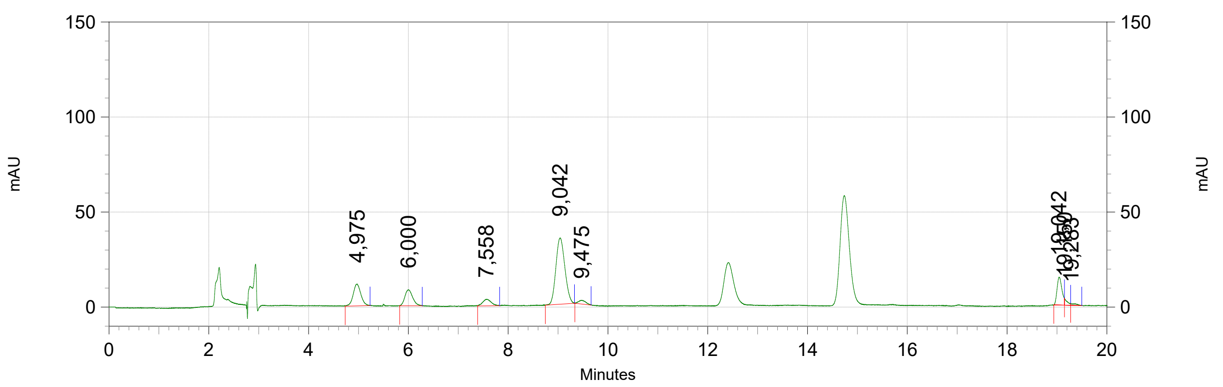

Det 168-440nm

Name	Retention Time	Area	Area Percent
Neoxanthin	4.975	122389	14.729
Violaxanthin	6.000	91221	10.978
Anteraxanthin	7.558	40777	4.907
Lutein	9.042	435877	52.457
Zeaxanthin	9.475	19865	2.391
Alfa carotene	19.042	99511	11.976
Beta carotene	19.150	13770	1.657

12

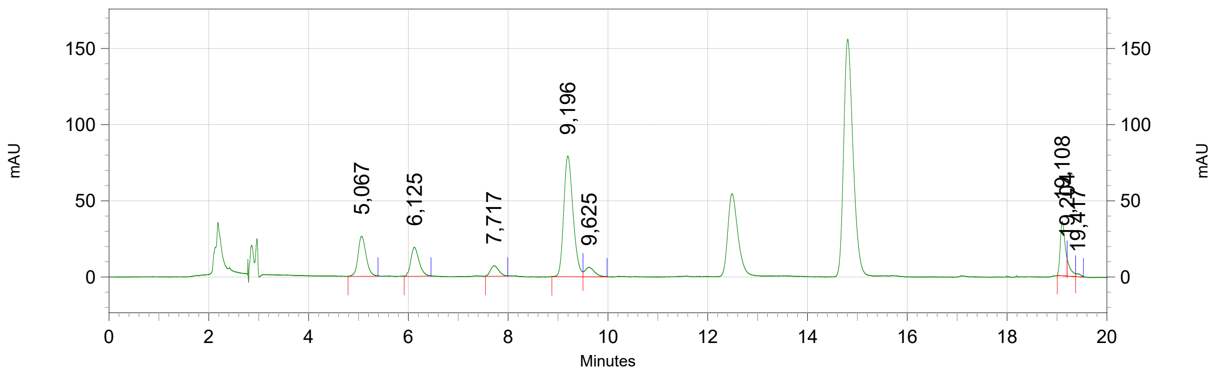

Det 168-440nm

Name	Retention Time	Area	Area Percent
Neoxanthin	5.067	293826	14.831
Violaxanthin	6.125	215572	10.881
Anteraxanthin	7.717	77173	3.895
Lutein	9.196	1024795	51.727
Zeaxanthin	9.625	79204	3.998
Alfa carotene	19.108	234126	11.818
Beta carotene	19.204	45784	2.311

15

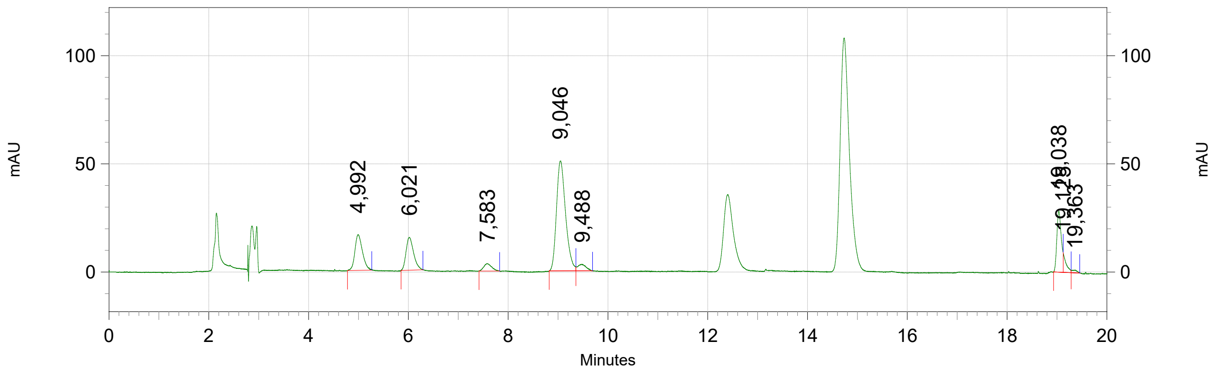

Det 168-440nm

Name	Retention Time	Area	Area Percent
Neoxanthin	4.992	174517	13.767
Violaxanthin	6.021	159682	12.597
Anteraxanthin	7.583	37852	2.986
Lutein	9.046	643504	50.763
Zeaxanthin	9.488	34514	2.723
Alfa carotene	19.038	172461	13.605
Beta carotene	19.125	35721	2.818

21

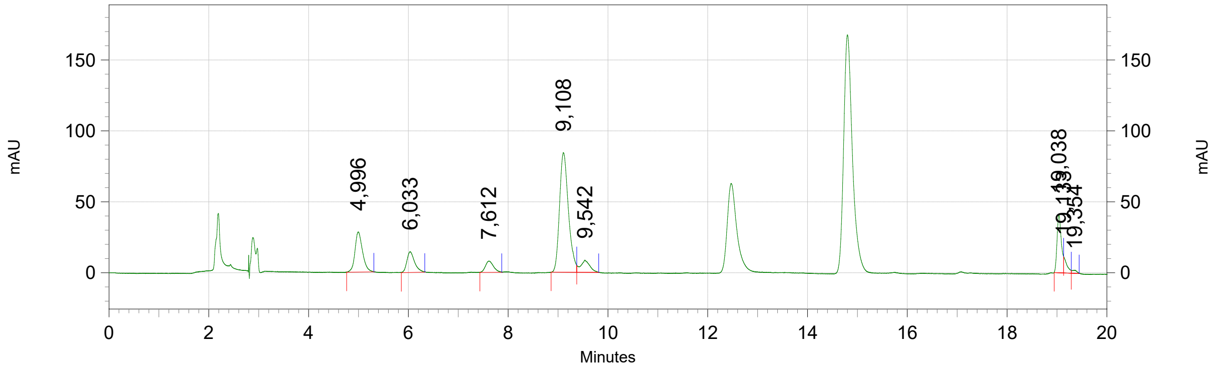

Det 168-440nm

Name	Retention Time	Area	Area Percent
Neoxanthin	4.996	295837	14.909
Violaxanthin	6.033	154489	7.786
Anteraxanthin	7.612	86467	4.358
Lutein	9.108	1038344	52.330
Zeaxanthin	9.542	106579	5.371
Alfa carotene	19.038	240881	12.140
Beta carotene	19.133	49351	2.487
